# A Cytokine Signalling Network for the Regulation of Inducible Nitric Oxide Synthase Expression in Rheumatoid Arthritis

**DOI:** 10.1371/journal.pone.0161306

**Published:** 2016-09-14

**Authors:** Poulami Dey, Venugopal Panga, Srivatsan Raghunathan

**Affiliations:** 1 Institute of Bioinformatics and Applied Biotechnology (IBAB), Biotech Park, Electronics City Phase I, Bengaluru 560 100, Karnataka, India; 2 Manipal University, Manipal, 576104, Karnataka, India; Georgia Regents University Cancer Center, UNITED STATES

## Abstract

In rheumatoid arthritis (RA), nitric oxide (NO) is implicated in inflammation, angiogenesis and tissue destruction. The enzyme inducible nitric oxide synthase (iNOS) is responsible for the localised over-production of NO in the synovial joints affected by RA. The pro- and anti-inflammatory cytokines stimulate the synovial macrophages and the fibroblast-like synoviocytes to express iNOS. Therefore, the cytokine signalling network underlying the regulation of iNOS is essential to understand the pathophysiology of the disease. By using information from the literature, we have constructed, for the first time, the cytokine signalling network involved in the regulation of iNOS expression. Using the differential expression patterns obtained by re-analysing the microarray data on the RA synovium and the synovial macrophages available in the Gene Expression Omnibus (GEO) database, we aimed to establish the role played by the network genes towards iNOS regulation in the RA synovium. Our analysis reveals that the network genes belonging to interferon (IFN) and interleukin-10 (IL-10) pathways are always up-regulated in the RA synovium whereas the genes which are part of the anti-inflammatory transforming growth factor-beta (TGF-β) signalling pathway are mostly down-regulated. We observed a consistent up-regulation of the transcription factor signal transducers and activators of transcription 1 (STAT1) in the RA synovium and the macrophages. Interestingly, we found a consistent up-regulation of the iNOS interacting protein ras-related C3 botulinum toxin substrate 2 (RAC2) in the RA synovium as well as the macrophages. Importantly, we have constructed a model to explain the impact of IFN and IL-10 pathways on Rac2-iNOS interaction leading to over-production of NO and thereby causing chronic inflammation in the RA synovium. The interplay between STAT1 and RAC2 in the regulation of NO could have implications for the identification of therapeutic targets for RA.

## Introduction

Rheumatoid arthritis (RA) is a systemic, polygenic, auto-immune inflammatory joint disease affecting about 1% of the world population [1]. The disease usually attacks the diarthrodial joints of hands, feet, shoulder and knee. The aetiology of the disease is still unknown [2]. RA is characterized by chronic synovitis. Early synovitis can meet with one of the three fates: resolving synovitis, non-RA persistent synovitis or RA synovitis. Histopathological studies on synovial tissues have failed to characterize the early synovitis which could subsequently develop into RA synovitis [3]. The well-established association of cytokines with inflammation prompted an experimental investigation and characterization of differential expressions of cytokines in the affected synovial fluid and tissues, including blood, in different groups of patients. Mainly, immunoassays and PCR-based techniques are used to quantify the presence of cytokines and their mRNAs respectively in the sample tissues. We have summarized the results on relative change in the cytokine concentrations in the affected tissues of pre-RA and RA patients published in literature (Table 1).

**Table 1:** The summary of the changes in the cytokine concentrations in pre-RA and RA patients. The “pre-RA patients” indicates the individuals who are in a very early stage of RA or lack the symptoms of RA at the time of study but eventually are diagnosed with RA.

Cytokines	Tissue	Pre-RA patients	RA patients
IL-1β (pro-inflammatory)	Serum [4–6]	**▲** (Compared to healthy controls)^a^	**▲** (Compared to healthy controls)
	Synovial Fluid [4]	N.A.^b^	**▲** (Compared to non-inflammatory arthritis)
TNF-α (pro-inflammatory)	Serum [5–7]	**▲** (Compared to healthy controls)	**▲** (Compared to healthy controls)
IL-6 (pro-inflammatory)	Serum [4, 6, 7]	**▲** (Compared to healthy controls)	**▲** (Compared to healthy controls)
	Synovial Fluid [4]	N.A.	▲ (Compared to non-inflammatory arthritis)
_IFN_-γ (pro-inflammatory)	Serum [6]	**▲** (Compared to healthy controls)	**▲** (Compared to healthy controls)
	Synovial Fluid [3]	N.A.	Not detected
TGF-β (anti-inflammatory)	Serum [5]	N.A.	**▼** (Compared to healthy controls)
	Synovial Fluid [8]	N.A.	**▼** (Compared to Psoriatic Arthritis)
IL-4 (anti-inflammatory)	Serum	**▲** (Compared to healthy controls) [6]	**▲**[6] ● [5] (Compared to healthy controls)
	Synovial Fluid	**▲** (Compared to healthy controls and RA patients) [3]	N.A.
IL-10 (anti-inflammatory)	Serum	**▼** [7]**▲** [6] (Compared to healthy controls)	**▼** [7] **▲** [6] ● [5] (Compared to healthy controls)

^a^ Symbols indicate significant increase (**▲**), decrease (**▼**) or no change (●) in cytokine concentrations of affected tissues from pre-RA and RA patients.

^b^N.A. implies no data available.

Table 1 corroborates a significant rise of the pro-inflammatory as well as some of the anti-inflammatory cytokines such as interleukin-4 (IL-4) and IL-10 in the serum of pre-RA and RA patients. The role of anti-inflammatory cytokines is to curb the pro-inflammatory cytokine responses. However, it is observed that RA synovitis prevails in spite of an increase in the concentration of anti-inflammatory cytokines. Conceivably, the concentration of anti-inflammatory cytokines may not be high enough to balance the pro-inflammatory responses, leading to RA synovitis.

The interplay of the pro- and anti-inflammatory cytokines induces the production of enzyme iNOS in the affected tissues of RA patients [1]. The enzyme iNOS catalyses the formation of nitric oxide (NO). Strong expression of iNOS has been observed in the synovium and cartilage of RA patients while the normal synovium from healthy individuals is completely devoid of it [9, 10]. Grabowski et al [9] have established that CD68+ macrophages of the synovial lining and fibroblasts are the source of iNOS expression in the RA synovium. Sakurai et al [10] have shown a strong correlation between the numbers of iNOS+ synoviocytes and the NO levels in the RA synovium. The concentrations of NO and nitrite, which is a stable metabolic form of NO, are significantly higher in the serum and the synovial tissues of RA patients than in healthy controls [11, 12]. The constitutively expressed nitric oxide synthases, endothelial NOS (eNOS) and neuronal NOS (nNOS), are involved in the maintenance of the physiological concentrations of NO. However, iNOS once induced by cytokines produces high concentrations of NO for a prolonged time period in affected RA tissues [13].

Ex-vivo experimental studies on human RA synoviocytes have shown that a combination of IL-1β, tumour necrosis factor-alpha (TNF-α) and interferon-gamma (IFN-γ) induces a significant increase in the production of iNOS and nitrite [9, 10, 14]. It is established that transforming growth factor-beta (TGF-β) and IL-4 down-regulate iNOS expression in murine macrophages [15, 16]. Active RA patients, on treatment with the IL-6 antagonist tocilizumab, show a prominent decrease in serum nitrite concentration [17]. It has been proposed that IL-10 is responsible for iNOS expression during the pathogenesis of RA [1].

To summarize, multiple studies on affected tissues of RA have established the following: (a) the existence of an imbalance in the expression of pro- and anti- inflammatory cytokines, (b) increased expression of iNOS and NO and (c) regulation of the expression of iNOS and consequently NO by various pro- and anti-inflammatory cytokines.

Wu et al have published a comprehensive molecular interaction map of RA consisting of 273 proteins and their associated genes [18]. However, the regulation of iNOS expression by cytokine signalling pathways in RA is not present in their network. In our study, in order to explain the role of downstream signalling molecules of various cytokine pathways that regulate iNOS in RA, we first constructed an elaborate signalling network of cytokines such as IL-1β, TNF-α, IL-6, IFN-γ, TGF-β, IL-4 and IL-10 using data on interactions between the signal-transducers reported in the literature. To the best of our knowledge, such a comprehensive cytokine signalling network regulating iNOS expression is created for the first time. Further, to establish the role of these network genes in RA, we have re-analysed the available microarray data on the disease. Our analysis reveals a possible mechanism that leads to the over-production of NO catalysed by iNOS in the RA synovium.

## Materials and Methods

### Construction of the cytokine signalling network

The cytokine signalling network which regulates the expression of iNOS in mammalian cells was constructed based on an extensive literature survey using PubMed. From the published results of experiments on rat, mouse and human cell lines, the required information on the regulation of iNOS were retrieved. We have used 25 search terms to retrieve 57 articles which were referred to construct the network. For example, the search term **(rheumatoid OR rheumatic OR arthritis OR arthritides) AND (cytokines OR cytokine OR cytokinin OR pathogenesis)** with a filter of “**Title”** for search fields was used in PubMed to retrieve references 1–8 in this study. Similarly, another 24 search terms were used to retrieve the articles related to the construction of the network. The complete list of the search terms and the corresponding retrieved references are included in S1 File. The information includes protein interactions, regulations and cross-talks between downstream signal transducers and transcription factors induced by cytokine stimulations [13, 16, 19–50]. Using this information, the cytokine signalling network regulating iNOS expression was constructed in CellDesigner 4.4. This network consists of proteins that are encoded by 106 genes. While constructing the network, a cell compartment enclosing a nucleus compartment was created. The nodes (proteins, protein complexes, receptors, mRNA and genes) were added in the appropriate compartments as given in the literature. The interactions (activation, inhibition, state transition, ubiquitination, transportation, transcription and translation) were then added to connect the nodes in the network. In case of an interaction where multiple molecules are responsible for the interaction process such as ubiquitination, the multiple lines are joined using “and” option in CellDesigner. The dotted lines were used to represent the interactions that were stated to be prevalent by unknown mechanism.

### Microarray data analysis

In GEO database, the search term **(Rheumatoid OR arthritis OR arthritides OR joint OR synovium OR synovial) AND (affymetrix)** was used with filters **Homo sapiens, Series and Expression profiling by array**. This search resulted in 156 hits. By reading the summary of each paper, we identified the studies which involved both RA synovial samples vs. normal controls and synovial macrophage samples vs. normal macrophage controls. This resulted in seven Affymetrix microarray datasets related to human RA patients (Table 2). Six of them correspond to synovial membrane and one corresponds to synovial macrophages. The analysis was carried out using the Bioconductor library in R statistical package [51]. For each of the seven datasets, the following analysis procedure was adapted: (i) The probe summarization was carried out using two of the standard normalization algorithms for Affymetrix data, namely MAS5 and RMA (ii) For each of the two summarization procedures, the differential expression of the genes between RA and control groups was computed using two sample independent t-test (iii) In order to reduce the false discovery rate, the p-values of the genes with fold changes greater than 0.5 or less than -0.5 in log2-scale were subjected to Benjamini-Hochberg (BH) correction. The genes which met the criterion of BH-corrected p-values < 0.1 were considered to be differentially expressed.

**Table 2:** Gene expression datasets used in this study.

Sample Origin	Sample Type and Number	GSE ID	Platform	Ref.
Synovial Tissue	5 RA, 5 Normal	GSE 1919	Affymetrix [HG-U95A]	[52]
Synovial Membrane	5 RA, 9 Normal	GSE7307	Affymetrix [HG-U133-Plus-2]	
Synovial Membrane	12 RA, 9 Normal [U133A], 12 RA, 4 Normal [U133B]	GSE12021	Affymetrix [HG-U133A] & Affymetrix [HG-U133B]	[53]
Synovial Membrane	13 RA, 10 Normal	GSE55457	Affymetrix [HG-U133A]	[54]
Synovial Membrane	10 RA, 10 Normal	GSE55235	Affymetrix [HG-U133A]	[54]
Macrophages	5 RA synovial fluid macrophages, 3 blood-derived normal macrophages	GSE10500	Affymetrix [HG-U95Av2]	[55]

In a given synovial data set, a gene was considered if it is differentially expressed in the same direction (up or down regulation) in both the normalization methods. In case a gene is differentially expressed in only one of the normalization methods and not in the other, it was considered only if it is expressed in the same direction in any other dataset in the complementary normalization method. For example, if a gene in dataset one expressed only in MAS5, it is required to be expressed in the same direction in any other dataset under RMA normalization.

Since there was only one macrophage data set, a gene was considered only if it is differentially expressed in the same direction (up or down regulation) in both the normalization methods.

In the resulting data, we looked at the 106 genes that are part of our cytokine network. For each gene, the number of datasets in which it was selected out of the six analysed was found.

The expression levels of the selected genes in the synovial and the macrophage datasets are presented in tabular form. In the case of synovial data, we have presented the maximum of the six up/down regulated values under each normalization.

## Results

### The cytokine signalling network regulating iNOS expression

**Fig 1:**
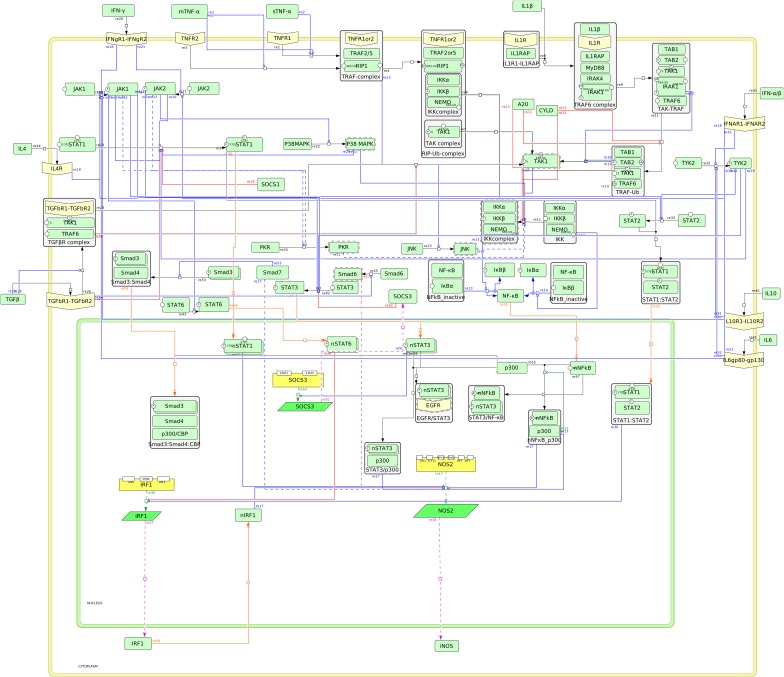
The cytokine signalling network regulating expression of iNOS. The network has two compartments; cytosol and nucleus. The nucleus is represented by the green-bordered box while the remaining network is within the yellow-bordered box representing cytosol. The yellow border represents the cell membrane. The network is constructed in CellDesigner and the following symbols therein are adapted: the light green rectangles are proteins, the green trapeziums are mRNAs, the yellow rectangles are genes, the pale yellow coloured structures on the cell membrane are receptors and the boxes with stacked proteins are the complexes. The blue lines ending with circles represent activation, brown lines ending with circles represent ubiquitination and the red lines represent inhibition. The black arrows denote state transition, the orange arrows denote transportation across compartments, the green arrows denote transcription and the pink arrows denote translation.

In this study, the signalling pathways of the cytokines leading to the regulation of the transcription factors or modulator proteins associated with iNOS expression are consolidated into a network (Fig 1). The cytokines TNF-α, IL-1β, IFN-γ, IL-10, IL-4, IL-6 and TGF-β are involved in the activation of transcription factors such as NF-κB, STAT1, IRF1, STAT3, STAT6, Oct-1, C/EBPβ and AP-1 [16, 23–25, 31]. The promoter region of human iNOS shows multiple binding sites for these transcription factors [13, 19, 34]. The cytokine signalling network includes a) NF-κB activation by TNF-α, IL-1β, IFN-γ and TGF-β, b) STAT1 activation by TGF-β, IFN, TNF-α and IL-1β, c) STAT1, STAT3 and STAT6 activation by IL-10, IL-6 and IL-4, d) regulation of Smad proteins by TGF-β e) activation of p300, C/EBPβ, AP-1 and Oct-1 by IFN-γ, TNF-α and IL1-β and f) regulation of iNOS expression by the signal transducers. The detailed description of the constructed signalling network regulating iNOS expression is included in S2 File.

### Role of the network genes towards iNOS regulation in RA synovium from gene expression data

The results from the differential expression analysis of microarray datasets on RA synovial tissue are used to explain the functional roles of the network genes played in the regulation of iNOS as described below.

#### Differential expression of STAT1 and IFN, IL-10 and IL-6 signalling genes in the RA synovium

Cytokines such as IFN-γ, IFN-α, IFN-β, IL-10 and IL-6 are involved in the activation of STAT1. The maximum fold changes of the pathway genes from our microarray data analysis are tabulated in Tables 3 and 4. The analysis of the RA synovial datasets leads to the following three insights.

**Table 3:** The maximum fold changes of the pathway genes corresponding to TNF-α, IL-1β pathways and few other transcription factors from our microarray data analysis.

	SYNOVIAL DATASETS			
Genes	Up Fold Change (on log2 scale)		Down Fold Change (on log2 scale)	
	MAS5	RMA	MAS5	RMA
FADD	0.87	0	-1	-0.71
CASP8	1.16	1.25	0	0
TNFRSF1B	1.36	0.92	0	0
BIRC3	2.34	2.07	0	0
TRAF5	0.74	0.72	0	0
RELA	0	0	-0.55	-0.5
NFKB1	0	0	-0.66	-0.58
IKBKB	0	0	-1.6	-0.6
NFKBIA	0	0	-1.36	-1.32
TNFAIP3	1.72	1.78	-1.01	0
CYLD	1.04	0.87	0	0
RNF11	0	0	-0.77	-0.74
IL1B	1.1	0.5	-0.99	-0.5
IL1RN	1.45	1.2	0	0
IL1R1	1.14	0	-2.89	-1.7
IL1RAP	0	0	-0.93	-1.11
MYD88	0.56	0.7	0	0
PELI1	0	0	-1.28	-1.12
PELI2	0	0	-1.4	-1.12
IRAK4	0.61	0.6	0	0
MAPK14	0	0	-0.77	-0.63
TAB3	0.57	0.66	0	0
POU2F1	0	0	-0.68	-0.68
CEBPB	0	0	-0.88	-0.9

**Table 4:** The maximum fold changes of the pathway genes corresponding to IFN, IL-10, IL-6, TGF-β pathways and AP-1 transcription factor from our microarray data analysis.

	SYNOVIAL DATASETS			
Genes	Up Fold Change (on log2 scale)		Down Fold Change (on log2 scale)	
	MAS5	RMA	MAS5	RMA
IFNA1	1.12	0.99	0	0
IFNB1	0	0	0	0
IFNAR1	0.9	0.57	0	0
IFNAR2	1.2	1.36	0	0
JAK2	1.67	1.13	0	0
TYK2	1.18	0.83	0	0
STAT1	2.81	3.15	0	0
STAT2	1.21	1.03	0	0
IRF1	1.31	1.43	0	0
IRF8	1.03	1.19	0	0
IRF9	0.7	0.66	0	0
IL10RA	1.36	1.18	0	0
IL10RB	1.03	0.87	0	0
SOCS1	1.48	1.13	0	0
IL6	0	0	-2.39	-2.6
IL6ST	0	0	-1.17	-0.8
STAT3	0	0	-0.54	-1.4
EGFR	0	0	-1.91	-2.22
SOCS3	0.81	0	-2.44	-2.28
PTPN6	1.64	1.14	0	0
PTPN11	0	0	-1.6	-1.04
FOS	0	0	-2	-2.04
JUND	0	0	-0.98	-2.09
FOSL2	0	0	-2.73	-2.27
TGFB1	2.53	1.94	0	0
LTBP1	0	0	-1.04	-0.69
LTBP2	0.69	0.72	0	0
TGFBR2	0	0	-2.95	-1.29
THBS1	0	0	-1.31	-1.65
SMAD2	1.08	0.72	-0.53	-0.7
SMAD3	0.88	0.79	-1.07	-1.03
SMAD7	0	0	-1.25	-1.2
NFE2L1	0	0	-1.04	-0.81

**Fig 2:**
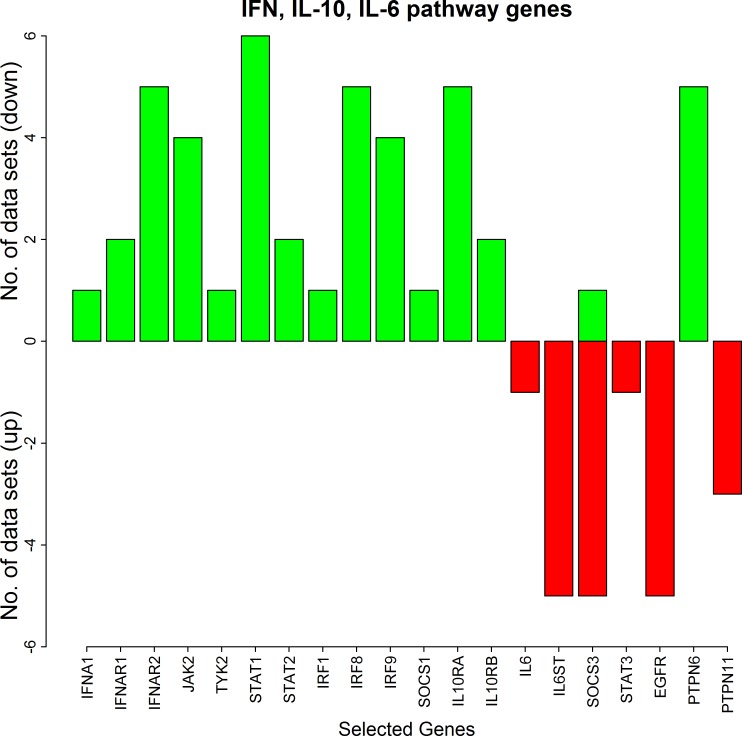
The number of the synovial datasets in which the pathway genes corresponding to IFN, IL-10, IL-6 pathways are differentially expressed. The green bars represent up-regulation of the genes in the datasets whereas the red bars represent their down-regulation.

(i) Consistent up-regulation of STAT1 in the RA synovium: The up-regulation of STAT1 in the RA synovium is experimentally observed by Kraan et al [56]. In agreement with this, our analysis confirmed an increased level of STAT1 in all the synovial datasets (Fig 2). The maximum fold-change of STAT1 up-regulation was observed as 3.15 in log2 scale (Table 4).

(ii) Consistent up-regulation of IFN and IL-10 pathway genes in the RA synovium: Besides STAT1, the other transcription factors like STAT2, IRF1, IRF9 and IRF8 which are part of IFN-mediated iNOS expression are up-regulated in at least one or more RA synovial datasets (Fig 2). Moreover, the JAK kinases JAK2 and TYK2 are also up-regulated in the synovial datasets. Further, the IFN type-I receptors and IL-10 receptors are up-regulated in two or more synovial datasets. The only IFN cytokine gene that is up-regulated is IFNA1. These genes which mediate IFN type-I, type-II and IL-10 signalling are never down-regulated in any of the synovial datasets. Additionally, SOCS1 which is induced by transcription factors STAT1 and IRF1, is also never down-regulated. The fold-changes of these genes in log2 scale are given in Table 4.

(iii) Consistent down-regulation of IL-6 pathway genes in the RA synovium: The gene for IL-6 receptor (IL6ST) is down-regulated in five synovial microarray datasets. The cytokine IL-6 and the transcription factor STAT3 are down-regulated in at least one dataset (Fig 2). EGFR which enhances iNOS expression by binding to STAT3 is also down-regulated in five datasets. None of these genes is up-regulated in any of the synovial datasets. SOCS3, the feedback inhibitor of IL-6 signalling is mostly down-regulated in five datasets and up-regulated in one dataset.

Thus the results show an increased expression of genes in IFN and IL-10 pathways and decreased expression of genes in IL-6 pathway (Fig 2). As described earlier, IFN, IL-10 and IL-6 cytokines are involved in the activation of STAT1. The increased expression of IFN and IL-10 pathway genes suggests that STAT1 is likely to be activated by IFN and IL-10 pathways in the RA synovium.

#### Differential expression of TGF-β signalling genes in the RA synovium

**Fig 3:**
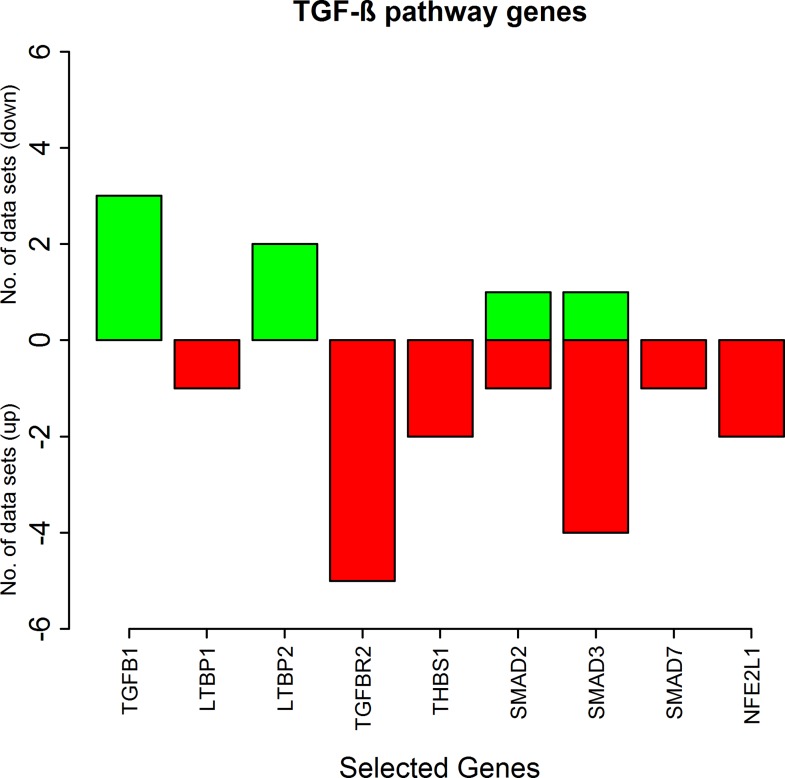
The number of the synovial datasets in which the pathway genes corresponding to TGF-β pathway are differentially expressed. The green bars represent up-regulation of the genes in the datasets whereas the red bars represent their down-regulation.

Most of the genes of the TGF-β pathway are down-regulated in the RA synovium (Fig 3). The maximum fold changes of these genes across all the synovial datasets are reported in Table 4. The genes TGFBR2, THBS1, SMAD7, TCF11 and LTBP1 are down-regulated in at least one dataset and are not up-regulated in any of the synovial datasets. The modulator proteins, Smad3 is up-regulated in one dataset and is down-regulated in four datasets whereas Smad2 is up-regulated in one dataset and is down-regulated in one dataset. TGFB1 and LTBP2 are up-regulated in three and two datasets respectively.

It is known that TGF-β and Smad3 facilitate inhibition of iNOS expression in RA [15, 20]. In this study, we have observed an up-regulation of the anti-inflammatory cytokine TGF-β (TGFB1). However, the downstream signal transducers of this pathway are mostly down-regulated. The transcription factor TCF11 which represses iNOS expression is also down-regulated. The decreased expression of the anti-inflammatory TGF-β pathway genes suggest that iNOS repression by TGF-β is not very prominent in the RA synovium.

#### Differential expression of TNF-α and IL-1β signalling genes in the RA synovium

**Fig 4:**
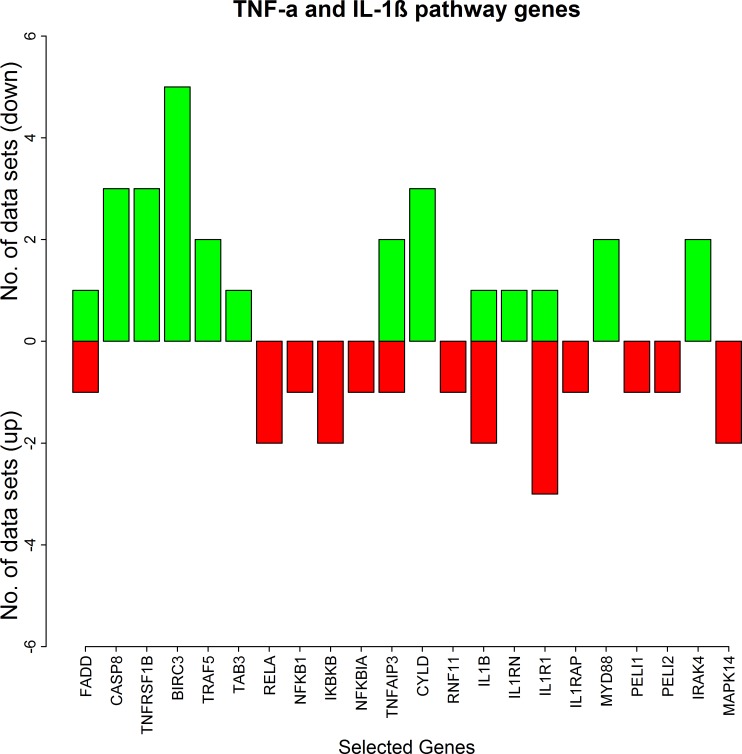
The number of the synovial datasets in which the pathway genes corresponding to TNF-α and IL-1β pathways are differentially expressed. The green bars represent up-regulation of the genes in the datasets whereas the red bars represent their down-regulation.

The results of our microarray data analysis show a mixed regulation (up- and down-) of the genes involved in the TNF-α and IL-1β signalling pathways (Fig 4 and Table 3). The feedback regulator TNFAIP3 (A20) which negatively regulates TNF-α and IL-1β signalling is up-regulated in two datasets and is down-regulated in one dataset. Another feedback regulator of TNF-α dependent NF-κB activation, CYLD is up in three datasets and is never down in any of the datasets. Additionally, the IκB kinase gene IKKB and the NF-κB genes NFKB1 (p50) and RELA (p65) are down-regulated.

#### Differential expression of other transcription factors in the RA synovium

**Fig 5:**
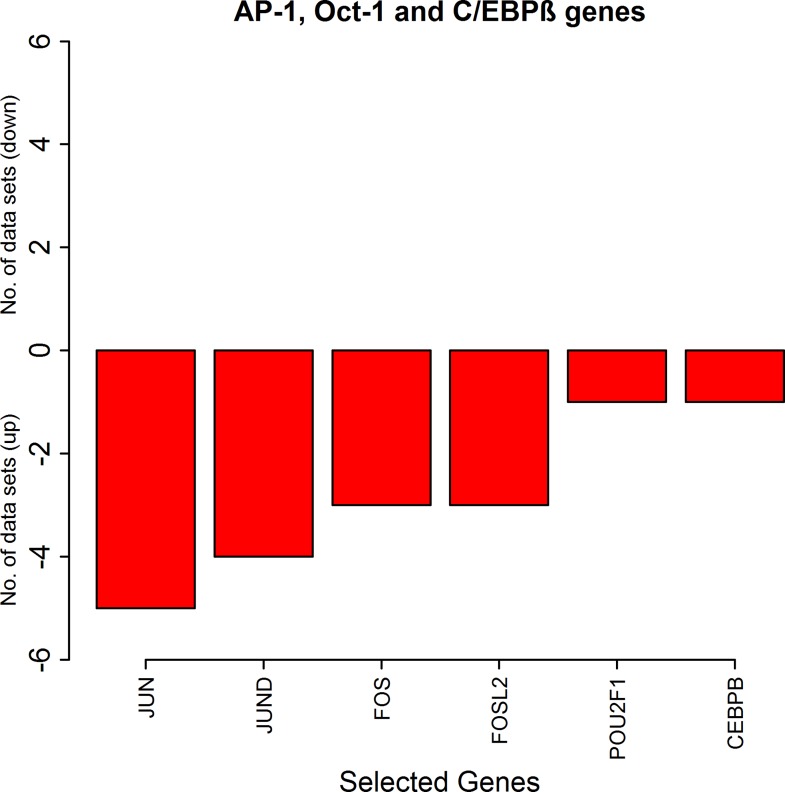
The number of the synovial datasets in which the pathway genes corresponding to transcription factors AP-1, Oct-1 and C/EBPβ are differentially expressed. The green bars represent up-regulation of the genes in the datasets whereas the red bars represent their down-regulation.

The genes which are part of the transcription factor AP-1, FOS, FOSL2 (Fra2), JUN and JUND are down-regulated in at least three or more synovial datasets and are never up in any of the datasets. The genes of the other transcription factors Oct-1 and C/EBPβ which are involved in enhancement of iNOS expression are down in the RA synovial datasets (Fig 5, Tables 3 and 4).

#### Differential expression of the network genes in the RA macrophages

The results of our microarray data analysis on the macrophage dataset in presented in Table 5. In contrast to the expression status in synovial datasets, the genes that were up-regulated in the macrophage dataset are janus kinase JAK1, feedback regulator SOCS1, IL10 receptor IL10RB, TGF-β signal modulators SMAD3 and SMAD7, the kinase p38MAPK and the transcription factor C/EBPβ. In spite of these differences, the transcription factor STAT1 which is up-regulated in the synovial dataset is also up-regulated in the macrophage dataset.

**Table 5:** The maximum fold changes of the pathway genes in macrophage dataset from our microarray data analysis.

	MACROPHAGE DATASET			
Genes	Up Fold Change (on log2 scale)		Down Fold Change (on log2 scale)	
	MAS5	RMA	MAS5	RMA
TNFRSF1B	1.62	1.02	0	0
RIPK1	0	0	-0.97	-0.83
MAP3K3	0	0	-1.63	-1.58
CFLAR	2.42	1.49	0	0
MAPK14	1.14	1.15	0	0
CEBPB	0.92	0.82	0	0
TAX1BP1	0.83	0.7	0	0
TAB1	0	0	-0.95	-0.89
TNFAIP3	1.91	1.83	0	0
IL1B	0	0	-0.81	-0.68
IL1R2	2.05	1.68	0	0
JAK1	1.4	1.32	0	0
STAT1	1.12	1.58	0	0
SOCS1	0	0	-0.9	-0.69
FOS	1.77	0.81	0	0
IL10RB	0	0	-1.46	-1.21
IL6ST	0	0	-0.84	-0.75
STAT6	0	0	-1.45	-0.95
SMAD3	0.89	0.8	0	0
SMAD4	0	0	-0.63	-0.53
SMAD7	0.82	0.98	0	0

#### Effects of medical therapies received by RA patients on gene expression data

**Fig 6:**
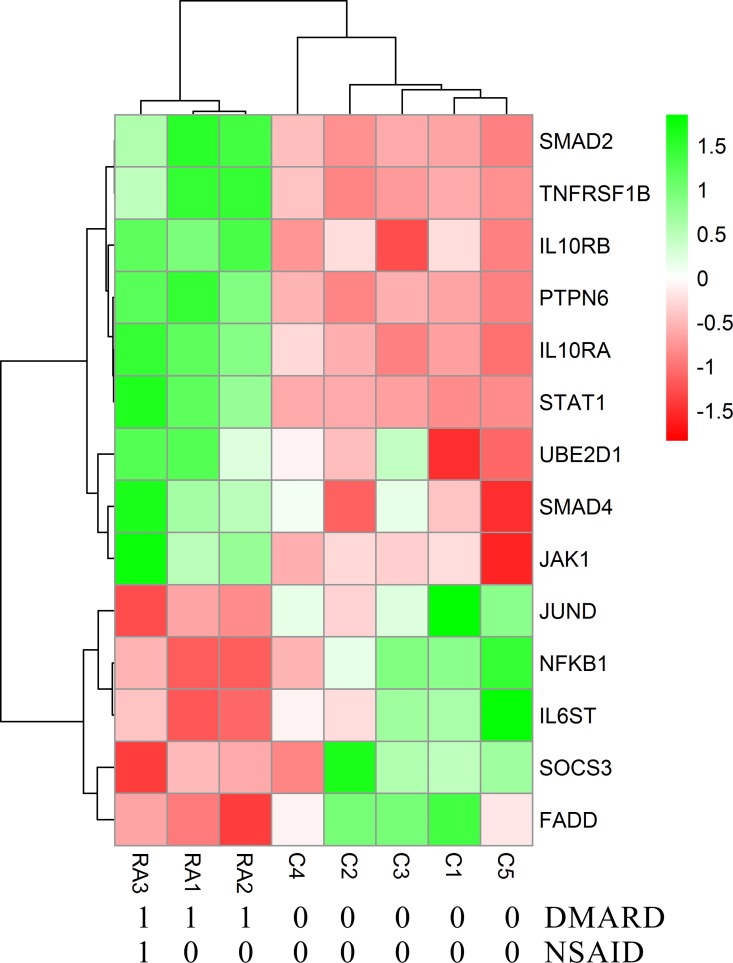
Hierarchical clustering of the individual samples based on the expressions of the network genes in GSE1919. In this dataset, three RA patients and five controls were considered for the clustering after eliminating two pooled RA samples. The RA and the control samples clustered into separate groups. The medical therapies received by the patients are indicated in the figure.

**Fig 7:**
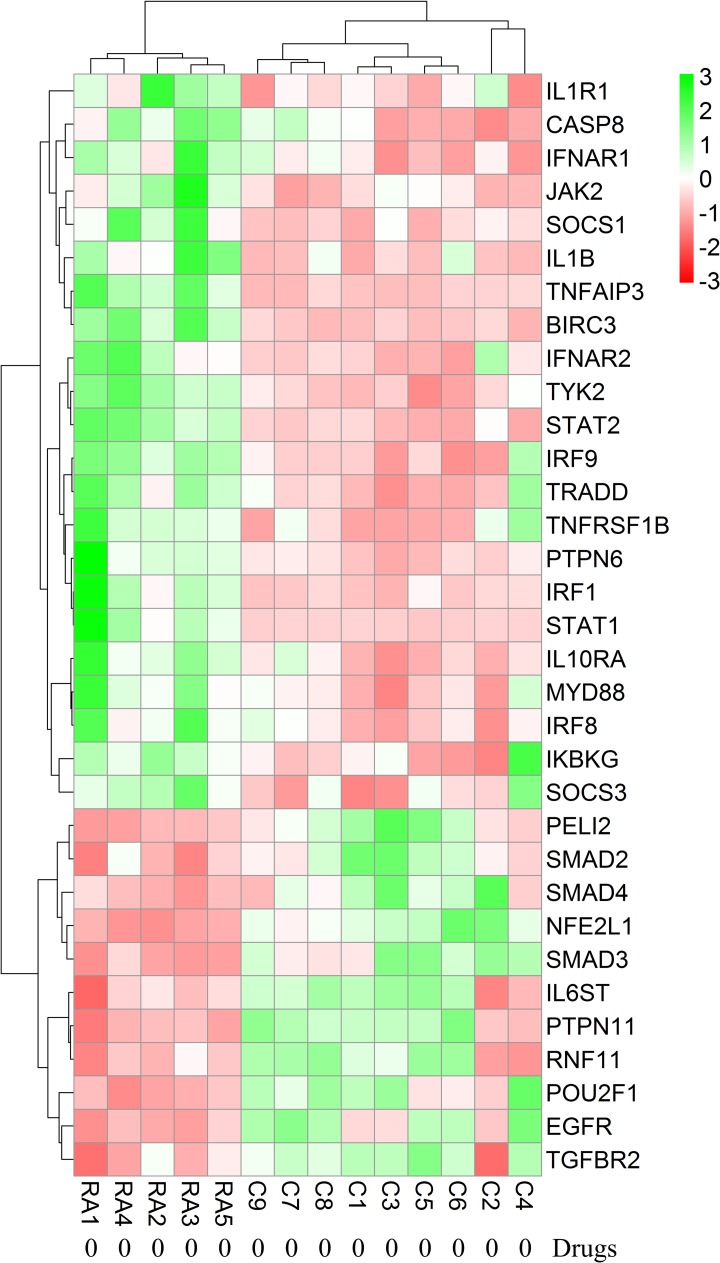
Hierarchical clustering of the individual samples based on the expressions of the network genes in GSE7307. In this dataset, five RA patients and nine controls were considered for the clustering. The RA and the control samples clustered into separate groups.

**Fig 8:**
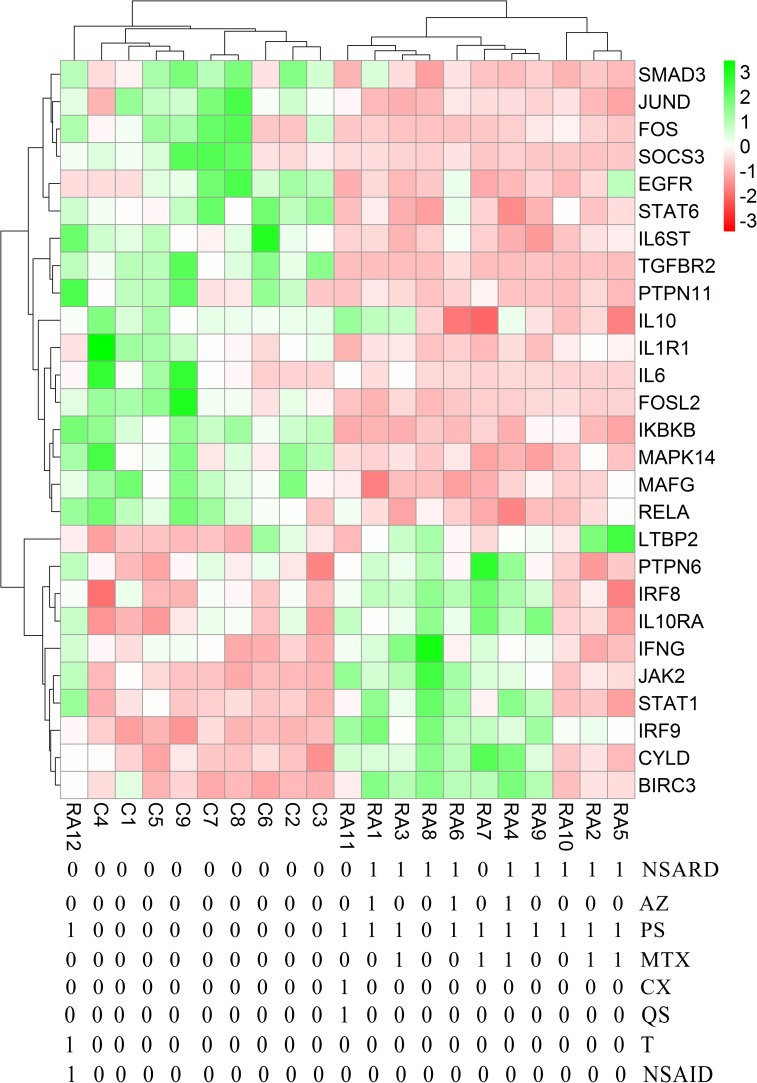
Hierarchical clustering of the individual samples based on the expressions of the network genes in GSE12021 (U133 A). In this dataset, twelve RA patients and nine controls were considered for the clustering. One RA sample clustered together with the control samples whereas the other RA samples clustered separately. The medical therapies received by the patients are indicated in the figure. The medical therapies received by the patients are non-steroidal anti-rheumatic drug (NSARD), Azulfidine (AZ), Prednisolone (PS), Methotrexate (MTX), Cox-2 inhibitor (CX), Quensyl (QS), Tilidin (T) and non-steroidal anti-inflammatory drug (NSAID).

**Fig 9:**
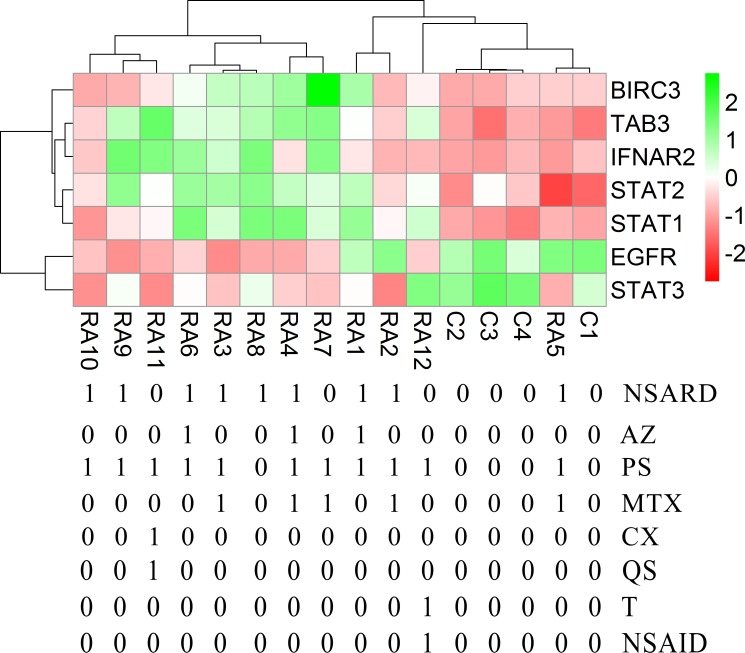
Hierarchical clustering of the individual samples based on the expressions of the network genes in GSE12021 (U133 B). In this dataset, twelve RA patients and four controls were considered for the clustering. One RA sample clustered together with the control samples. The medical therapies received by the patients are indicated in the figure. The medical therapies received by the patients are non-steroidal anti-rheumatic drug (NSARD), Azulfidine (AZ), Prednisolone (PS), Methotrexate (MTX), Cox-2 inhibitor (CX), Quensyl (QS), Tilidin (T) and non-steroidal anti-inflammatory drug (NSAID).

**Fig 10:**
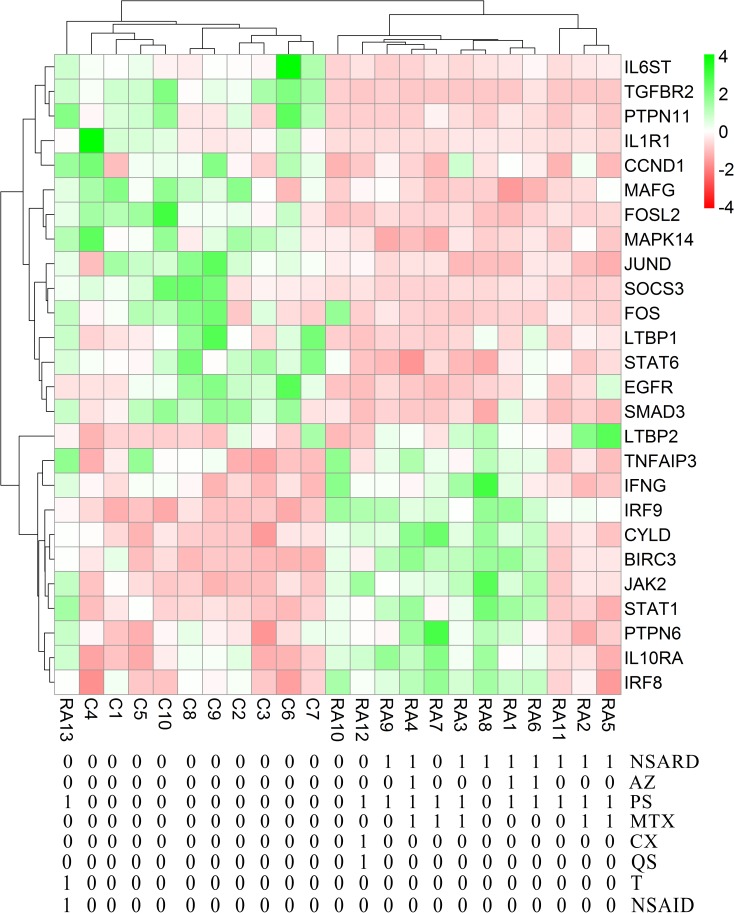
Hierarchical clustering of the individual samples based on the expressions of the network genes in GSE55457. In this dataset, thirteen RA patients and ten controls were considered for the clustering. One RA sample clustered together with the control samples whereas the other RA samples clustered separately. The medical therapies received by the patients are indicated in the figure. The medical therapies received by the patients are non-steroidal anti-rheumatic drug (NSARD), Azulfidine (AZ), Prednisolone (PS), Methotrexate (MTX), Cox-2 inhibitor (CX), Quensyl (QS), Tilidin (T) and non-steroidal anti-inflammatory drug (NSAID).

**Fig 11:**
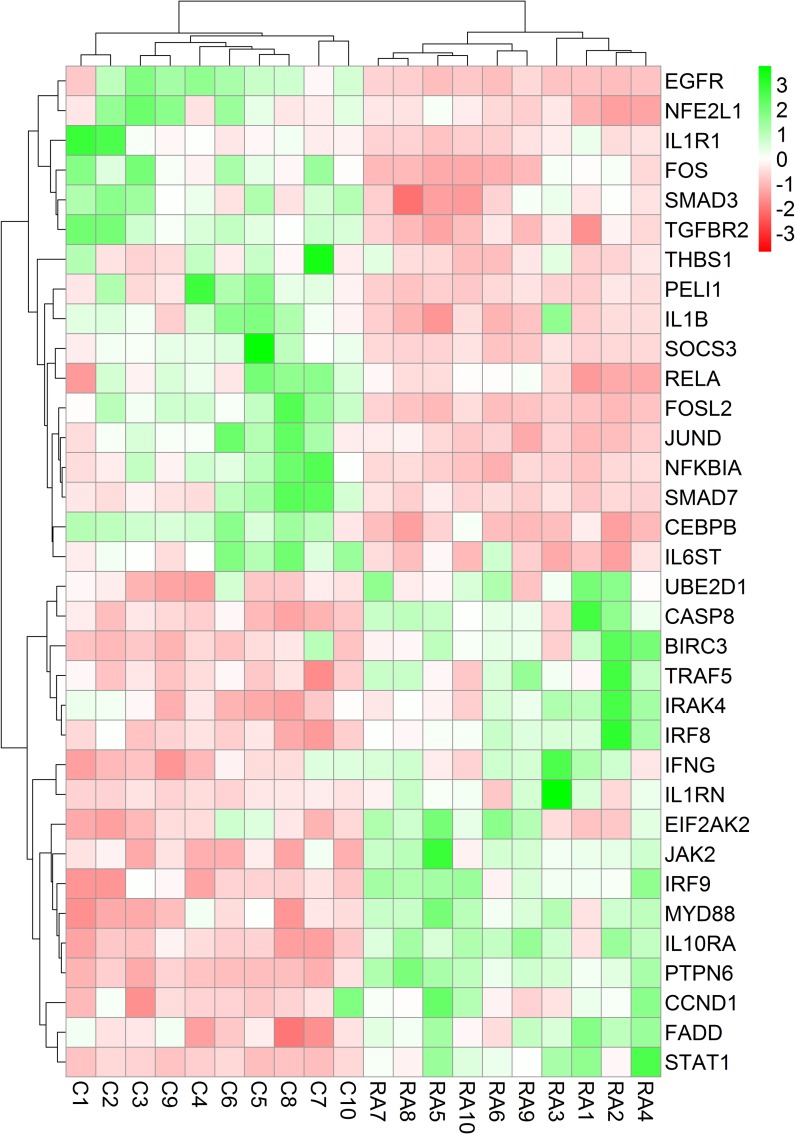
Hierarchical clustering of the individual samples based on the expressions of the network genes in GSE55235. In this dataset, ten RA patients and ten controls were considered for the clustering. The RA and the control samples clustered into separate groups. The medical therapies received by the RA patients are not specified in this dataset.

**Fig 12:**
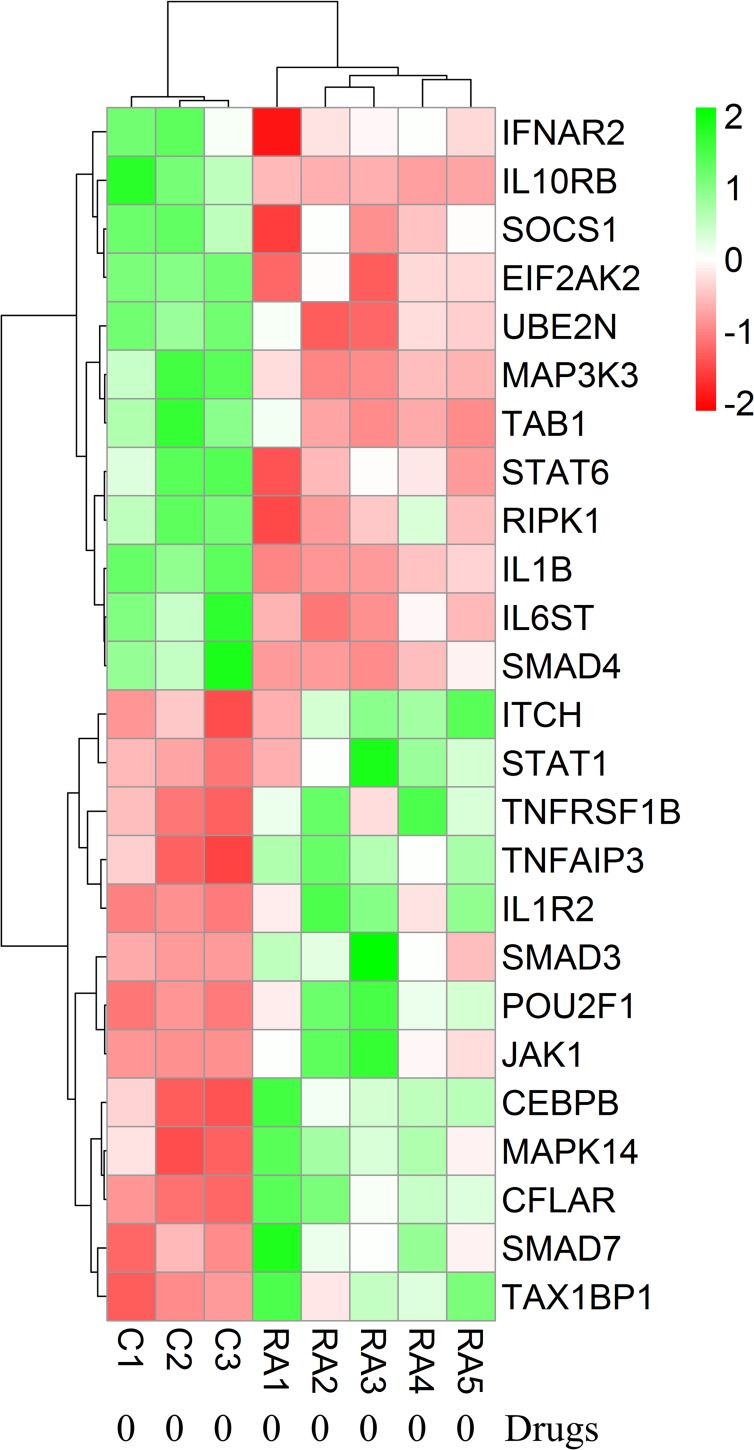
Hierarchical clustering of the individual samples based on the expressions of the network genes in GSE10500. In this dataset, five RA patients and three controls were considered for the clustering. The RA and the control samples clustered into separate groups.

Each RA patient belonging to different synovial and macrophage datasets underwent different combinations of medical therapies. The medical therapies received by the patients are disease modifying anti-rheumatic drug (DMARD), non-steroidal anti-rheumatic drug (NSARD), Azulfidine (AZ), Prednisolone (PS), Methotrexate (MTX), Cox-2 inhibitor (CX), Quensyl (QS), Tilidin (T) and non-steroidal anti-inflammatory drug (NSAID).The details of the medical therapies are listed in Table 6. It is seen that within a dataset, some patients received same combination of medical therapies whereas others received different combinations. To test if the gene expressions are influenced by these medical therapies, the samples treated with different combinations of medical therapies were hierarchically clustered based on the expression of the network genes in these samples. Except for GSE1919, it is observed that the gene expressions are not influenced by the type of the medical therapies received by the RA patients (Figs 6–12).

**Table 6:** Medical therapies received RA patients.

Dataset	Patients	Medical Therapies
GSE1919	RA1	DMARD
	RA2	DMARD
	RA3	DMARD + NSAID
GSE7307	RA1	no therapy used
	RA2	no therapy used
	RA3	no therapy used
	RA4	no therapy used
	RA5	no therapy used
GSE12021A	RA1	NSARD + Azulfidine + Prednisolone
	RA2	NSARD + MTX + Prednisolone
	RA3	NSARD + MTX+ Prednisolone
	RA4	NSARD + Azulfidine + Prednisolone + MTX
	RA5	NSARD + MTX + Prednisolone
	RA6	NSARD + Azulfidine + Prednisolone
	RA7	MTX + Prednisolone
	RA8	NSARD
	RA9	NSARD + Prednisolone
	RA10	NSARD + Prednisolone
	RA11	COX-2 inhibitor + Prednisolone + Quensyl
	RA12	NSAID + Tilidin + Prednisolone
GSE12021B	RA1	NSARD + Azulfidine + Prednisolone
	RA2	NSARD + MTX + Prednisolone
	RA3	NSARD + MTX+ Prednisolone
	RA4	NSARD + Azulfidine + Prednisolone + MTX
	RA5	NSARD + MTX + Prednisolone
	RA6	NSARD + Azulfidine + Prednisolone
	RA7	MTX + Prednisolone
	RA8	NSARD
	RA9	NSARD + Prednisolone
	RA10	NSARD + Prednisolone
	RA11	COX-2 inhibitor + Prednisolone + Quensyl
	RA12	NSAID + Tilidin + Prednisolone
GSE55457	RA1	NSARD + Azulfidine + Prednisolone
	RA2	NSARD + MTX + Prednisolone
	RA3	NSARD + MTX+ Prednisolone
	RA4	NSARD + Azulfidine + Prednisolone + MTX
	RA5	NSARD + MTX + Prednisolone
	RA6	NSARD + Azulfidine + Prednisolone
	RA7	MTX + Prednisolone
	RA8	NSARD
	RA9	NSARD + Prednisolone
	RA10	no therapy used
	RA11	NSARD + Prednisolone
	RA12	COX-2 inhibitor + Prednisolone + Quensyl
	RA13	NSAID + Tilidin + Prednisolone
GSE55235	RA1	Therapies not mentioned
	RA2	Therapies not mentioned
	RA3	Therapies not mentioned
	RA4	Therapies not mentioned
	RA5	Therapies not mentioned
	RA6	Therapies not mentioned
	RA7	Therapies not mentioned
	RA8	Therapies not mentioned
	RA9	Therapies not mentioned
	RA10	Therapies not mentioned
GSE10500	RA1	no therapy used
	RA2	no therapy used
	RA3	no therapy used
	RA4	no therapy used
	RA5	no therapy used

### Proposed role of STAT1 in the over-production of NO in the RA synovium

**Fig 13:**
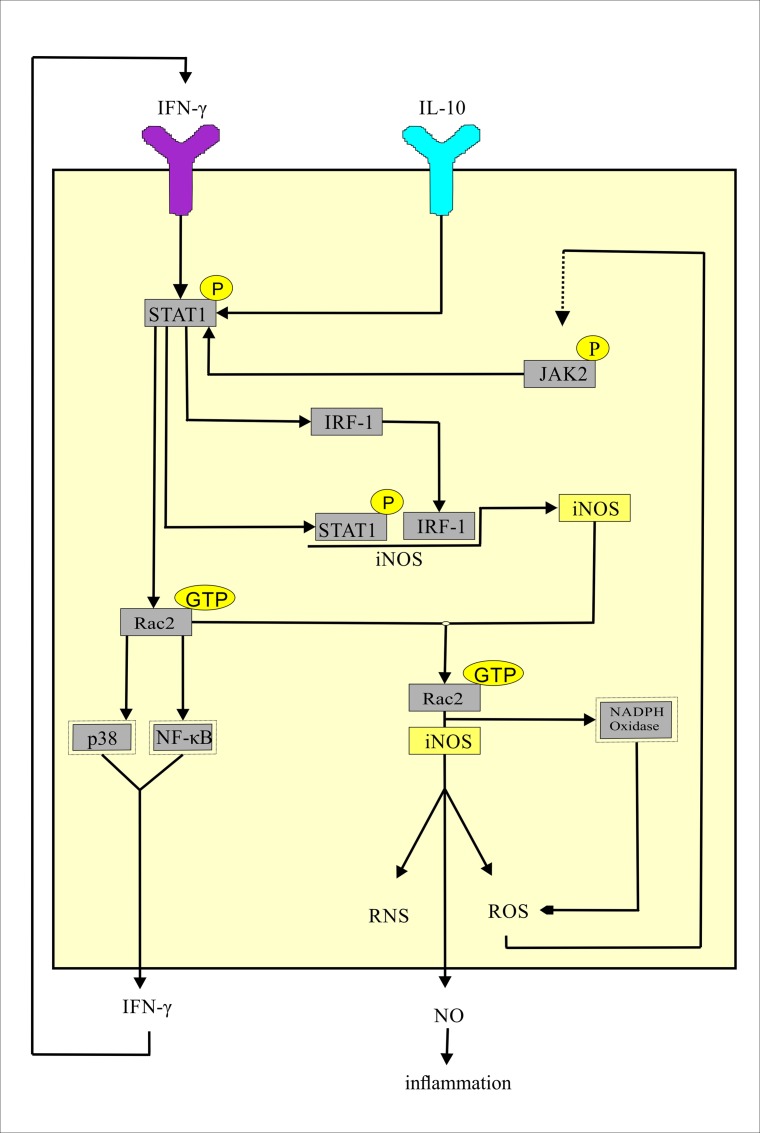
The proposed model by which IFN/ IL-10 pathway mediates chronic inflammation in the RA synovium by elevating NO. The coloured box with the black boundary is a representative cell in the RA synovium. The Y-shaped structures are the cytokine receptors and the rectangles within the cell are protein. The black arrows indicate either activation or production. The dotted black arrow indicates an unknown mechanism of JAK2 activation.

Our analysis confirmed an increased level of STAT1 in the synovium as well as the synovial macrophages of RA patients. Therefore we wanted to check if STAT1 interacts with any other iNOS interacting proteins that regulate the production of NO catalysed by iNOS. To find the answer, we have retrieved all the iNOS interacting proteins from three protein-protein interaction databases namely Pathway Commons, HPRD and InnateDB [57, 58, 59]. Among the iNOS-interacting proteins which are not part of the cytokine signalling network, only the RAC2 gene is up-regulated in both the macrophage and the synovial datasets. Hu et al [60] have shown that the activated STAT1 fixes Rac in a GTP-bound state (Rac-GTP) in macrophages. Rac2-GTP enhances the production of IFN-γ by inducing the IFN-γ promoter via activation of the transcription factors p38 and NF-κB [61]. The elevated expressions of STAT1 and RAC2 in our analysis demonstrate an increased possibility of Rac2 activation by activated STAT1 in the RA synovium and the synovial macrophages. This paves the way for the formation of a positive feedback loop resulting in an increased production of the pro-inflammatory cytokine IFN-γ and subsequent activation of STAT1 and Rac2 (Fig 13). The interaction between Rac2 and iNOS is crucial for Rac2 mediated NADPH oxidase activation. NADPH oxidase induces reactive oxygen species (ROS) production. ROS potentiates the expression of iNOS by activating JAK2/ IRF1 pathway [62]. Up-regulation of JAK2 and IRF1 observed in our analysis supports the possibility of ROS mediated expression of iNOS in the RA synovium. Thus, the activation of Rac2 by activated STAT1 also triggers the second positive feedback loop for enhanced production of ROS and iNOS in the RA affected synovium (Fig 13). Kuncewicz et al have stated that the balance between GTP- and GDP-bound Rac2 might play a role in the kinetics or stability of RAC2 interaction with iNOS [63]. It is experimentally shown that Rac2-GTP interacts with iNOS protein to generate high amounts of NO, ROS and reactive nitrogen species (RNS) in human cells [62]. NO induces a depletion of ATP leading to necrosis. Increased necrosis facilitates release of more antigen [64]. This leads to a chronic inflammation in the RA synovium. NO stimulates oxidative stress in the RA synovium. It reacts with superoxide to form peroxynitrite which is responsible for pro-inflammatory effects in the affected RA synovium. NO is also responsible for an up-regulation of the matrix metalloproteinases which are the key players for joint destruction in RA [65]. Thus, we have constructed a model of IFN/ IL-10 pathway mediated chronic inflammation via elevated levels of NO in the RA synovium.

## Discussion

In this study, we have constructed the cytokine signalling network underlying the regulation of iNOS in the RA synovium. The network includes seven cytokines among which TNF-α, IL-1β and IFN-γ are known to be pro-inflammatory and the remaining four cytokines namely IL-6, TGF-β, IL-10 and IL-4 are known to be pleiotropic in many diseases. But studies on RA have shown IL-6 mainly as pro-inflammatory whereas TGF-β, IL-10 and IL-4 as anti-inflammatory. As far as the regulation of iNOS is concerned, experiments in literature have shown the cytokines TGF-β and IL-4 are known to play contrasting roles. While constructing the network, although we have included certain relevant paths by which TGF-β can up- as well as down-regulate iNOS, the paths by which IL-4 up-regulates iNOS have not been included. We acknowledge this as a limitation in our network construction. Though the number of cytokines and the transcription factors included in the network is limited compared to the huge number of cytokines involved in the RA pathology, it is the most comprehensive network to date for describing the iNOS regulation.

Using the available microarray datasets on RA synovium and macrophages in GEO database, the differential expressions of the network genes are measured. The differential expressions are analysed to establish the functional roles of the network genes played in the regulation of iNOS. Most of the IFN and IL-10 pathway genes including the transcription factors STAT1, STAT2, IRF1 belonging to the network are always up-regulated in the RA synovium. An elevated expression of the transcription factor STAT1 is identified to be consistent in the RA synovium and the synovial macrophages. Additionally, the expressions of the most of the network genes belonging to the pathway signalled by the anti-inflammatory cytokine TGF-β are decreased in the RA synovium. The network genes belonging to TNF-α and IL-1β pathways are observed to be regulated in a mixed (up- and down-) nature.

We have observed that the expression of SOCS1, a negative regulator of JAK/STAT pathway is increased in the RA synovium. The published literature supports the activation of JAK/STAT pathway in RA [66, 67]. SOCS1, a negative regulator, is also one of the target genes of this pathway. SOCS1, being a target gene of the JAK/STAT pathway, is expected to be up-regulated while the pathway is active. In support of this, Isomaki et al have reported the up-regulation of SOCS1 in the RA synovial membrane over healthy controls [68]. The cytokines such as IFN, IL-10 and IL-6 activate the JAK/STAT pathway. IFN and IL-10 pathway genes such as the cytokine receptors, JAK2, TYK2, STAT1 and IRF1 are observed to be up-regulated in our analysis. Therefore it is highly likely that the JAK/STAT pathway is active in the RA synovium. In relation to this, in our analysis we have observed elevated expression of the STAT1 target genes (Table 7). This observation provides strong evidence that the JAK/STAT pathway is definitely active despite the up-regulation of SOCS1.

**Table 7:** Up-regulation of STAT1 target genes. The number of the synovial datasets in which the STAT1-target genes are up-regulated.

	No of synovial datasets in which the gene is up-regulated					
	1	2	3	4	5	6
STAT1						
GBP1						
ZC3HAV1						
GCH1						
BAK1						
ABCC4						
APOL1						
CTSS						
CXCL10						
CXCL9						
GBP2						
CYP1B1						
BCL2L11						
GBP5						
ICAM1						
IFI16						
IL7						
IRF1						
IRF7						
IRF9						
ITK						
LYN						
NMI						
PSMB8						
PSMB9						
SH3KBP1						
SOD2						
STAT2						
TAP1						
TAP2						
AIM2						
CIITA						
HLA-E						
IDO1						
C3						
MX1						
RNF19B						
APOL3						
IFI35						
WARS						
TGM2						

The pro-inflammatory cytokine IL-6 is usually elevated in RA affected synovial tissue. Our analysis shows no up-regulation of IL-6 in one of the datasets while it is not differentially expressed in the others. An experimental study by Chen et al confirmed no up-regulation of IL-6 in four out of seven and six out of eight synovial tissue and fluid samples from RA patients compared to healthy controls [69]. It is possible that in some cases IL-6 might not be up-regulated in the RA synovium. Additionally, IL-6R is not up-regulated in the analysis. IL-6 receptors are expressed by neutrophils, monocytes/macrophages and some lymphocytes which infiltrate the RA synovium exhibiting chronic inflammation. Though the resident synoviocytes do not express IL-6R, these cells carry forward the IL-6 signalling by an alternative trans-signalling pathway [70, 71]. Usually, the transition from acute to chronic inflammation in RA is controlled by IL-6. It is not known whether the synovium samples collected from the RA patients belonging to various data sets considered for this study manifested chronic inflammation. This is one of the limitations of this study.

The IL-6 receptor (gp130), on binding to IL-6, activates JAK1, JAK2 and TYK2. The JAKs promote activation of STAT3 which relocates to the nucleus to turn on STAT3-responsive genes. Earlier studies have shown the presence of activated STAT3 in the RA synovial tissues although the expression of STAT3 was not elevated [72]. In addition to JAK3, STAT3 can also be activated by the kinases JAK1 and TYK2 that are activated by IL-10 signalling pathway. In this analysis, we have observed an up-regulation of IL-10 pathway genes including TYK2. Though STAT3 is not up-regulated, it is likely that the phosphorylation of STAT3 is carried out by TYK2 in the RA synovium.

In this study, we have also constructed a model on the possible mechanisms by which IFN/IL-10 pathway drive chronic inflammation in the RA synovium. We have found that the expressions of RAC2, an iNOS-interacting protein is consistently up-regulated in the RA synovium. Interestingly, RAC1/RAC2 was identified to be an important hub in the analysis of the molecular interaction network of RA [18]. In this study, we have described the mechanism by which the STAT1 mediated RAC2 activation can lead to chronic inflammation in the RA synovium via an over-production of NO.

## Conclusion

In this study, a cytokine signalling network for the regulation of iNOS is created for the first time. From the results of the microarray data analysis on RA synovial datasets, we observed that the most of the genes from IFN- and IL-10 pathways regulating iNOS expression are always up-regulated whereas many genes from the TGF-β anti-inflammatory pathway are down-regulated. The transcription factor STAT1 is up-regulated in both the RA synovium and the synovial macrophages. It is concluded that STAT1 is also influencing the production of NO in the RA synovium by regulating iNOS interaction with RAC2. A model of IFN-γ/ IL-10 pathway mediated chronic inflammation via elevated levels of NO in the RA synovium has been constructed.

## Supporting Information

S1 Fig The cytokine signalling network regulating expression of iNOS. (TIF)

S2 Fig The activation of NF-κB by TNF-α, IL-1β, IFN-γ and TGF-β. (TIF)

S3 Fig The regulation of STAT1 phosphorylation at Y701 by TGF-β and the intermediate kinases of IFN. (TIF)

S4 Fig The activation of STAT1 phosphorylation at S727 by IFN-γ, IL-1β and TNF-α. (TIF)

S5 Fig The activation of STAT1, STAT3 and STAT6 by IL-10, 1L-6 and IL-4. (TIF)

S6 Fig The activation of Smad proteins by TGF-β. (TIF)

S7 Fig The activation of p300, C/EBPβ, AP-1 and Oct-1 by IFN-γ, TNF-α and IL1-β. (TIF)

S1 File Search terms used in PubMed for the retrieval of references used to construct the cytokine signalling network. (DOCX)

S2 File Description of the cytokine signalling network regulating iNOS expression. (DOCX)
