# Supplementary figures and images for: A Cytokine Signalling Network for the Regulation of Inducible Nitric Oxide Synthase Expression in Rheumatoid Arthritis
Source: PLoS One. 2016 Sep 14;11(9):e0161306. doi: 10.1371/journal.pone.0161306 (PMC5023176; doi:10.1371/journal.pone.0161306)

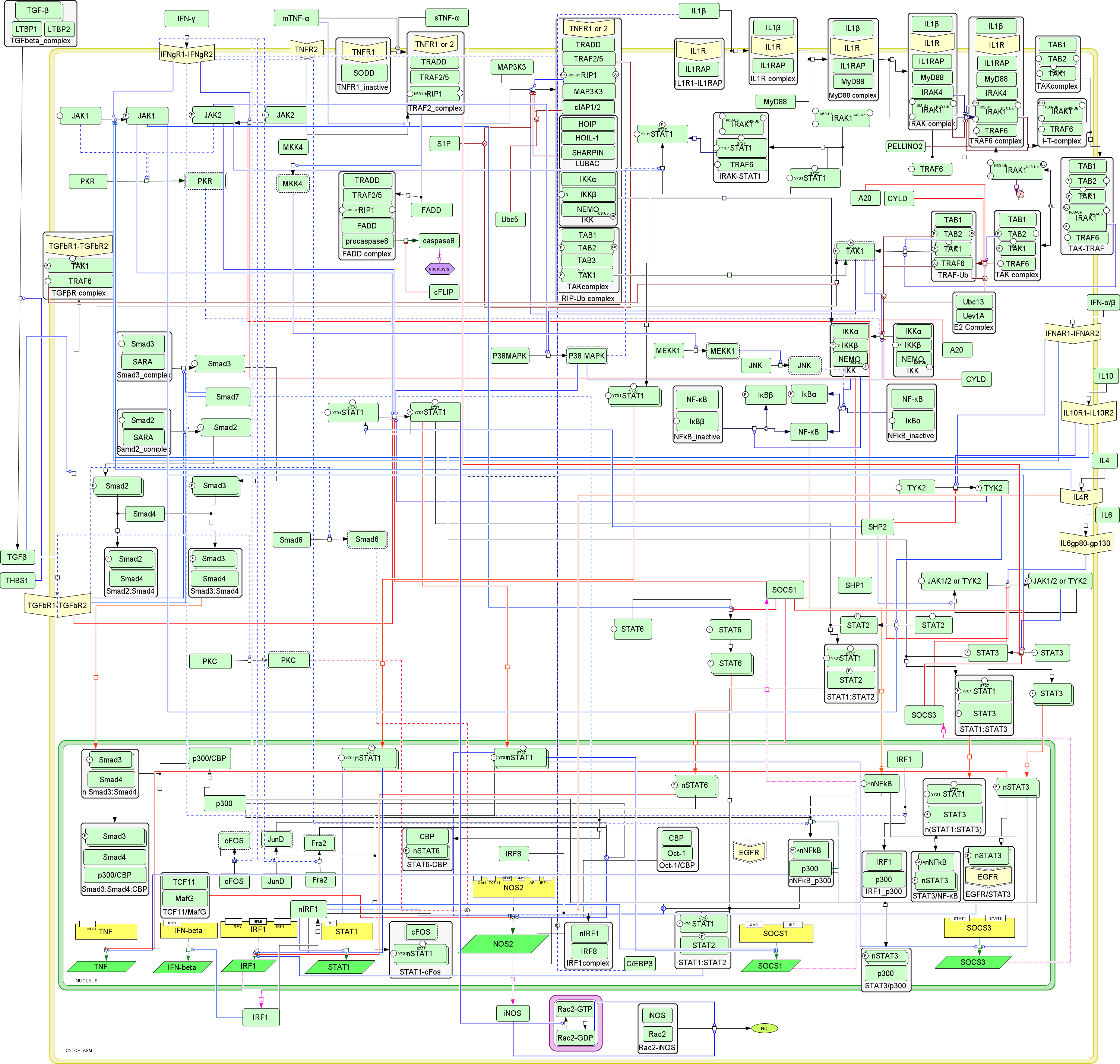

Supplement: S1 Fig — (TIF) [file pone.0161306.s001.tif]

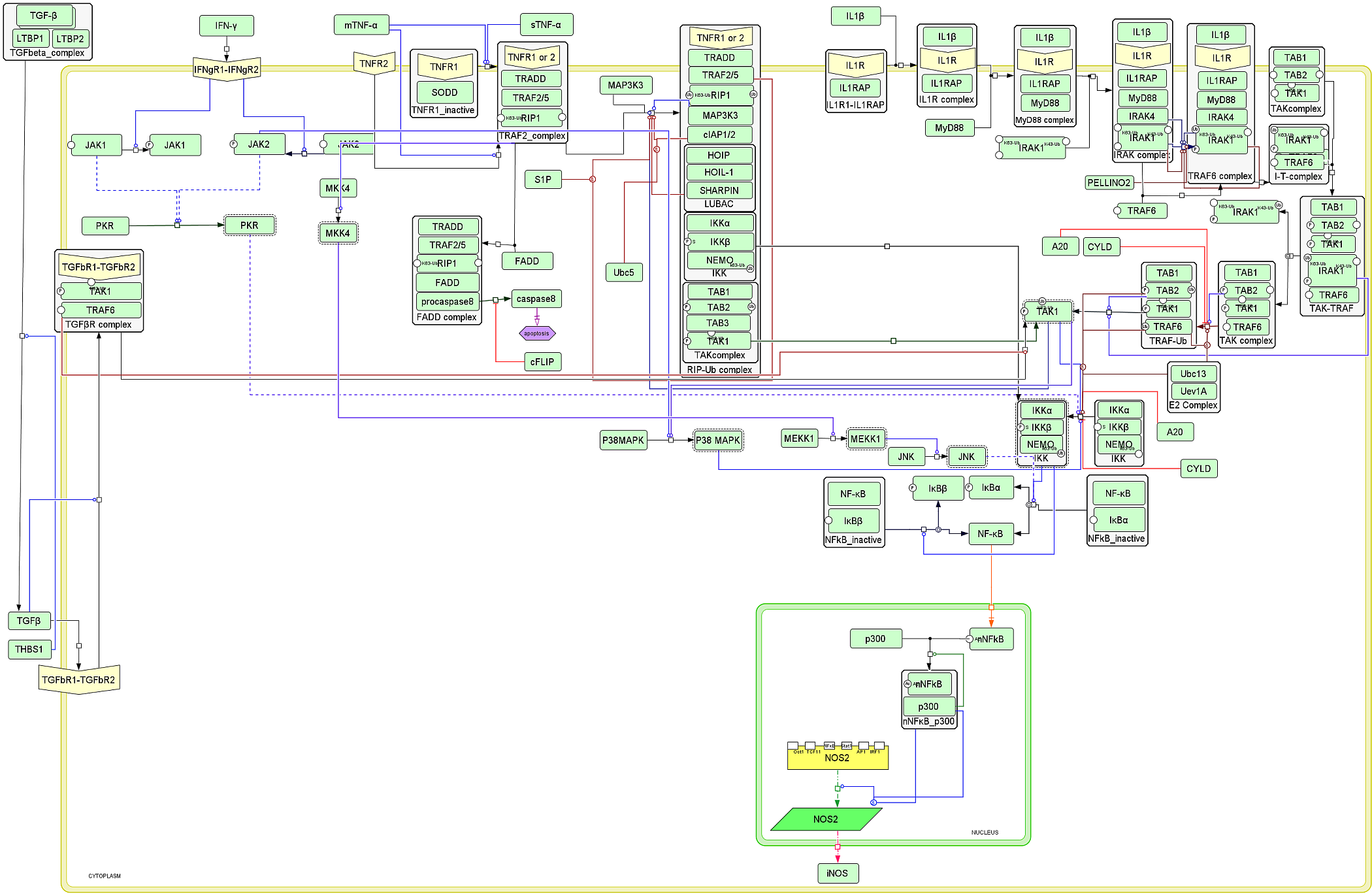

Supplement: S2 Fig — (TIF) [file pone.0161306.s002.tif]

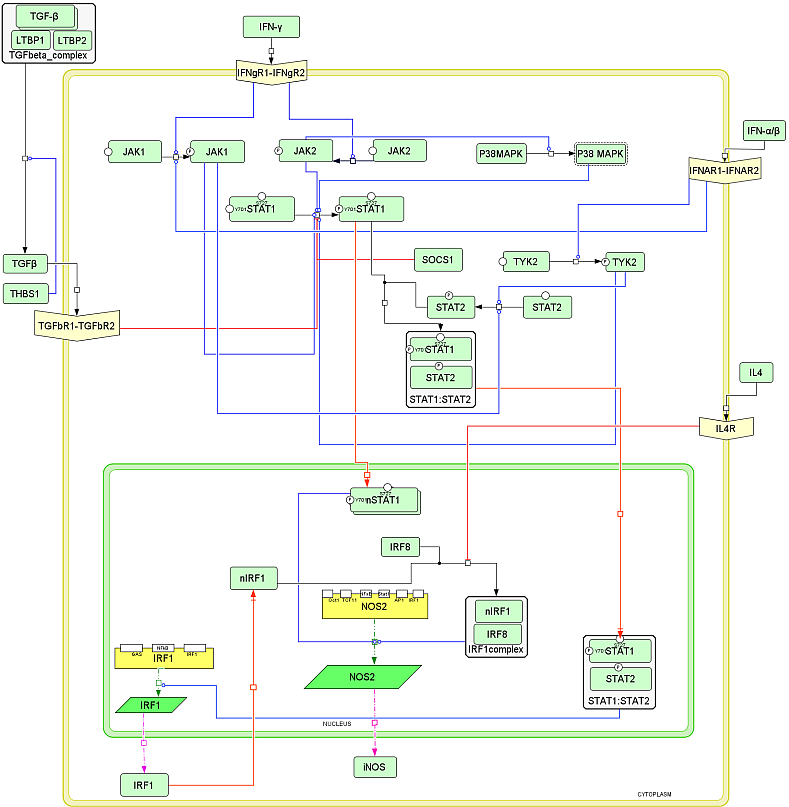

Supplement: S3 Fig — (TIF) [file pone.0161306.s003.tif]

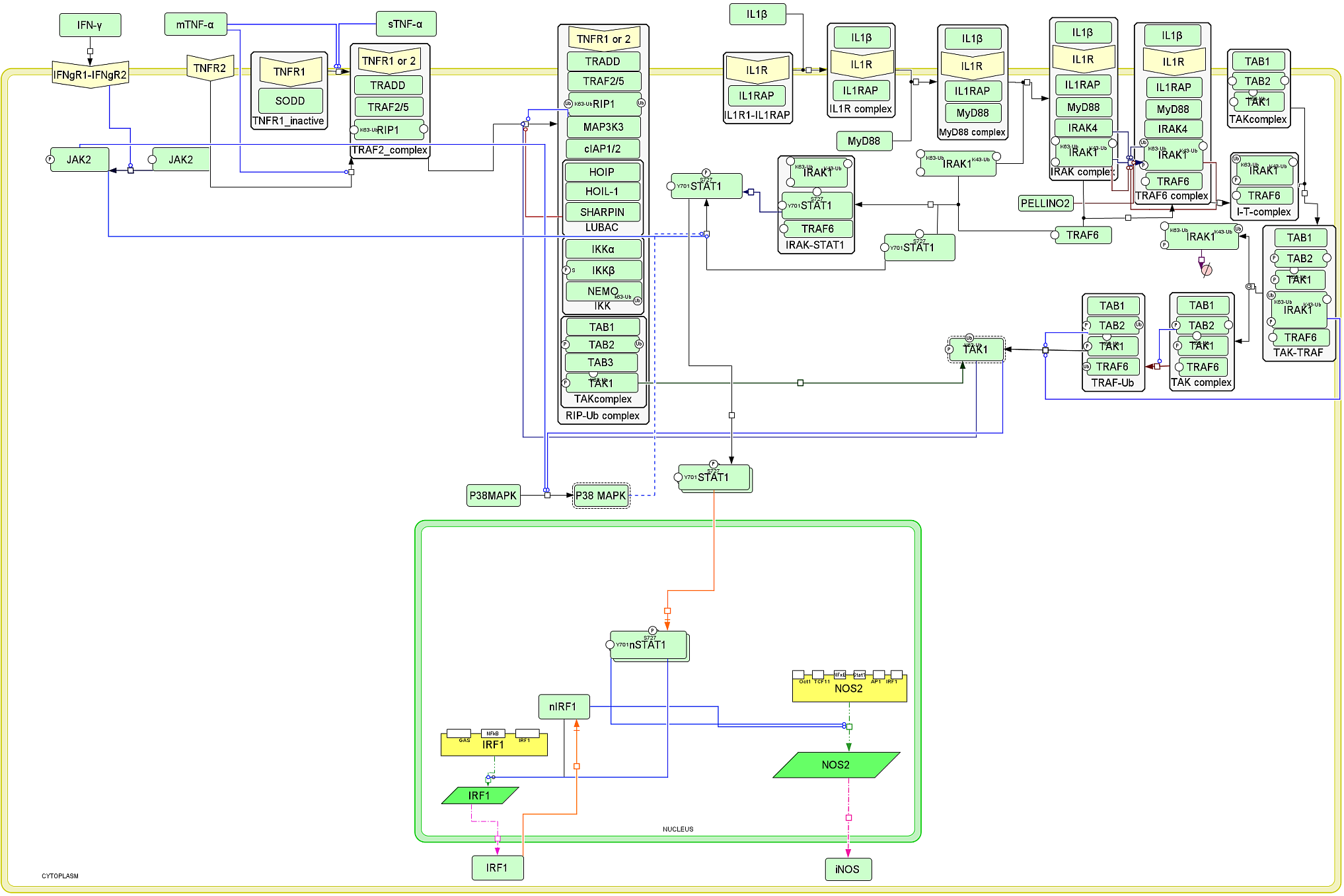

Supplement: S4 Fig — (TIF) [file pone.0161306.s004.tif]

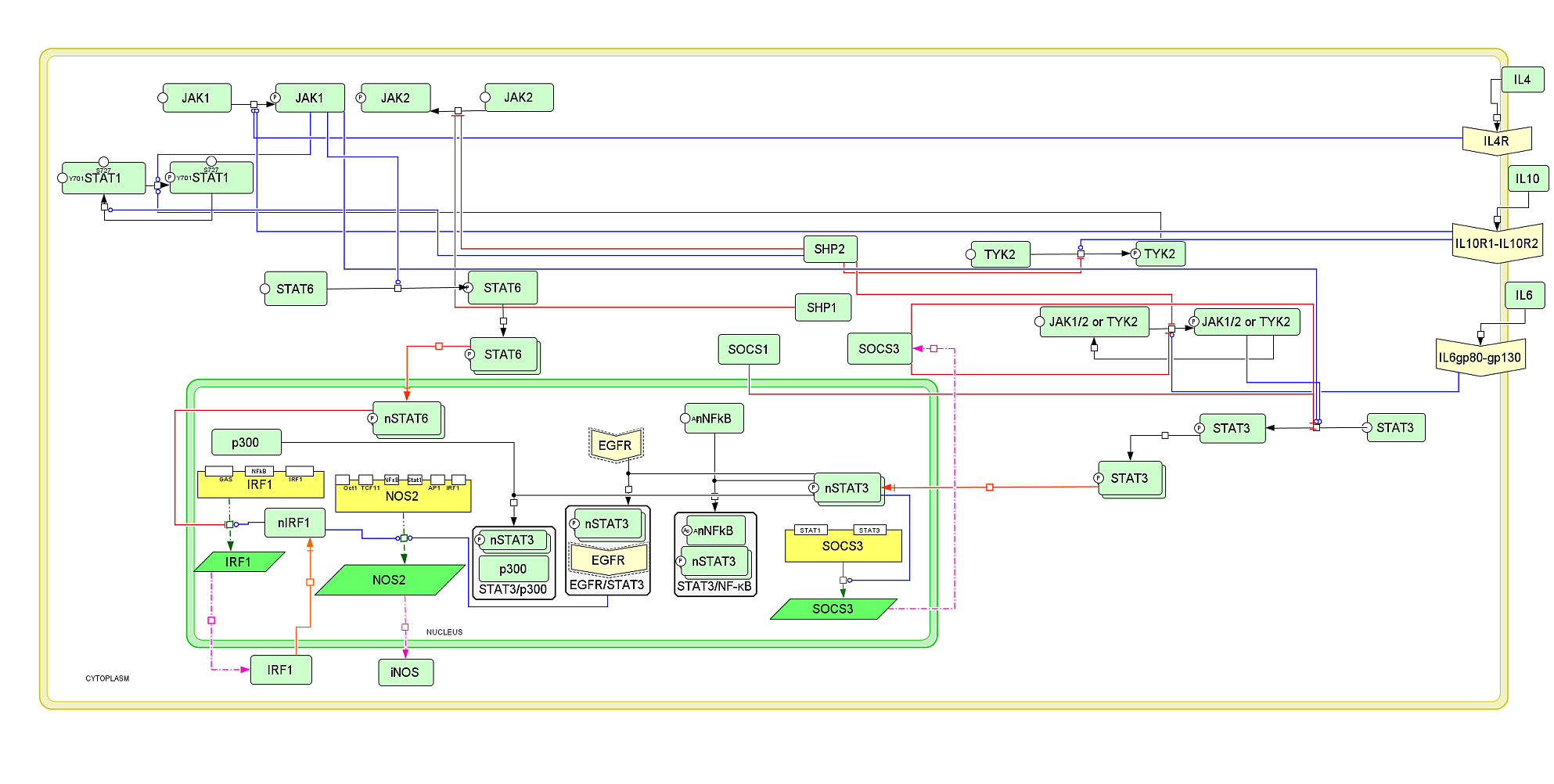

Supplement: S5 Fig — (TIF) [file pone.0161306.s005.tif]

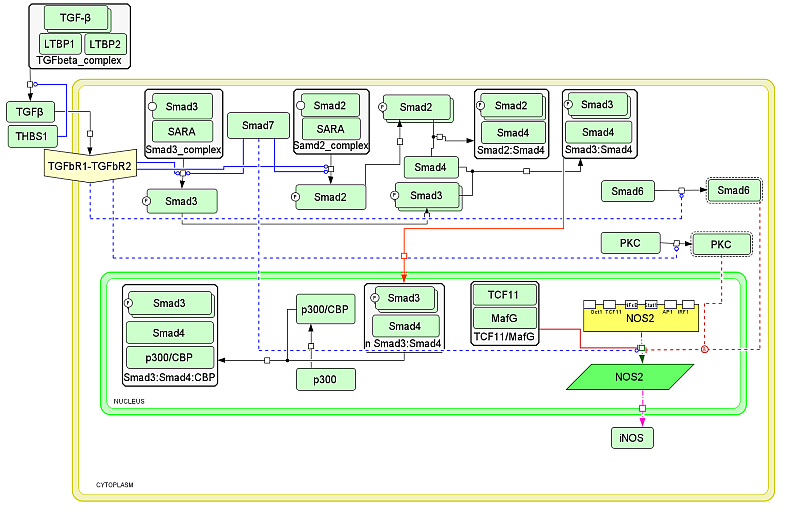

Supplement: S6 Fig — (TIF) [file pone.0161306.s006.tif]

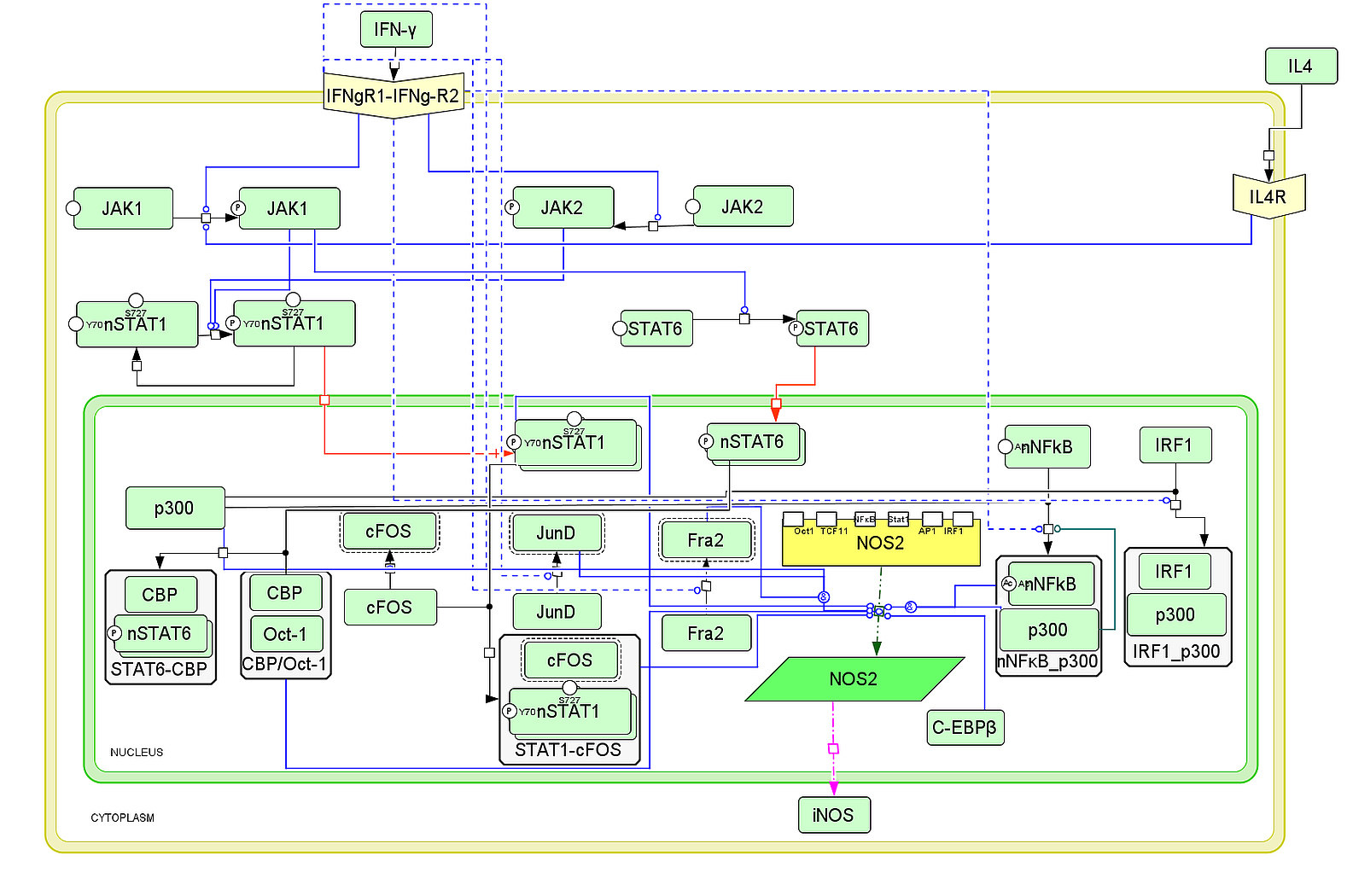

Supplement: S7 Fig — (TIF) [file pone.0161306.s007.tif]
