# Supplementary material for: A Cytokine Signalling Network for the Regulation of Inducible Nitric Oxide Synthase Expression in Rheumatoid Arthritis
Source: PLoS One. 2016 Sep 14;11(9):e0161306. doi: 10.1371/journal.pone.0161306 (PMC5023176; doi:10.1371/journal.pone.0161306)
Supplement: S1 File — (DOCX) [file pone.0161306.s008.docx]

**Search terms used in PubMed for the retrieval of references used to construct the cytokine signalling network**

1. **Search terms used for retrieving the list of cytokines and transcription factors regulating the expression of iNOS in Rheumatoid Arthritis**
2. (rheumatoid OR rheumatic OR arthritis OR arthritides) AND (cytokines OR cytokine OR cytokinin OR pathogenesis)

Search fields: Title

This search resulted in 2059 papers.

References 1, 2, 3, 4, 5, 6, 7, 8 were part of this search.

1. ("NOS2" OR "iNOS" OR "inducible nitric oxide synthase" OR "HEP-NOS" OR "NOS2A" OR "NOS-2" OR "Nanos2" OR "Nanos homolog 2" OR "inducible nitric oxide 2A" OR "Nitric Oxide Synthase Type II" OR "Inducible NOS Protein" OR "INOS Enzyme" OR "Nitric Oxide Synthase, Type II" OR "NOS-II" OR "NOS II" OR "Inducible Nitric Oxide Synthase" OR "Nitric Oxide Synthase II" OR "nitric oxide synthase-2” OR "inducible NO synthase") AND (rheumatoid OR rheumatic OR arthritis OR arthritides OR disease OR diseases)

Search fields: Title

This search resulted in 155 papers.

References 9, 10 and 19 were part of this search.

1. (nitrite OR nitrate OR nitric) AND (rheumatoid OR rheumatic OR arthritis OR arthritides OR joint OR synovium OR synovial)

Search fields: Title

This search resulted in 225 papers.

References 11, 12 and 14 were part of this search.

1. ("NOS2" OR "iNOS" OR "inducible nitric oxide synthase" OR "HEP-NOS" OR "NOS2A" OR "NOS-2" OR "Nanos2" OR "Nanos homolog 2" OR "inducible nitric oxide 2A" OR "Nitric Oxide Synthase Type II" OR "Inducible NOS Protein" OR "INOS Enzyme" OR "Nitric Oxide Synthase, Type II" OR "NOS-II" OR "NOS II" OR "Inducible Nitric Oxide Synthase" OR "Nitric Oxide Synthase II" OR "nitric oxide synthase-2" OR "inducible NO synthase") AND (regulation OR regulates OR regulate)

Search fields: Title

This search resulted in 500 papers.

References 13, 16, 25, 34, 37 and 38 were part of this search.

Based on the literature survey (i to iv mentioned above) we shortlisted the seven cytokines and the eight transcription factors which regulate the expression of iNOS in rheumatoid arthritis.

1. **Search terms used for collecting information on interactions in the cytokine network regulating the expression of iNOS**
2. ("Transforming Growth Factor" OR "Transforming Growth Factor beta" OR "TGFb" OR "Milk Growth Factor" OR "Factor, Milk Growth" OR "Growth Factor, Milk" OR "TGF-beta" OR "TGFbeta" OR "Platelet Transforming Growth Factor" OR "Bone-Derived Transforming Growth Factor" OR "Bone Derived Transforming Growth Factor") AND ("NOS2" OR "iNOS" OR "inducible nitric oxide synthase" OR "HEP-NOS" OR "NOS2A" OR "NOS-2" OR "Nanos2" OR "Nanos homolog 2" OR "inducible nitric oxide 2A" OR "Nitric Oxide Synthase Type II" OR "Inducible NOS Protein" OR "INOS Enzyme" OR "Nitric Oxide Synthase, Type II" OR "NOS-II" OR "NOS II" OR "Inducible Nitric Oxide Synthase" OR "Nitric Oxide Synthase II" OR "nitric oxide synthase-2” OR “inducible NO synthase”)

Search fields: Title

This search resulted in 29 papers.

References 20, 32, 44 were part of this search.

1. ("STAT1 Transcription Factor" OR "Transcription Factor, STAT1" OR "Transcription Factor STAT91" OR "STAT-91 Transcription Factor" OR "STAT 91 Transcription Factor" OR "Transcription Factor, STAT-91" OR "Signal Transducer and Activator of Transcription 1" OR "STAT-91 Protein" OR "STAT 91 Protein" OR "STAT91 Transcription Factor" OR "Transcription Factor, STAT91" OR "STAT1 Protein" OR "Gamma-Activated Factor, 91 kDa" OR "Gamma Activated Factor, 91 kDa" OR "STAT1" OR "STAT-1") AND ("gene expression")

Search fields: Title

This search resulted in 50 papers.

Reference 21 was part of this search.

1. ("STAT1 Transcription Factor" OR "Transcription Factor, STAT1" OR "Transcription Factor STAT91" OR "STAT-91 Transcription Factor" OR "STAT 91 Transcription Factor" OR "Transcription Factor, STAT-91" OR "Signal Transducer and Activator of Transcription 1" OR "STAT-91 Protein" OR "STAT 91 Protein" OR "STAT91 Transcription Factor" OR "Transcription Factor, STAT91" OR "STAT1 Protein" OR "Gamma-Activated Factor, 91 kDa" OR "Gamma Activated Factor, 91 kDa" OR "STAT1" OR "STAT-1" OR "STAT") AND ("tyrosine phosphatase" OR "tyrosine phosphatases")

Search fields: Title

This search resulted in 29 papers.

Reference 22 was part of this search.

1. ("Interleukin-10" OR "Interleukin 10" OR "IL10" OR "IL-10" OR "CSIF-10" OR "Cytokine Synthesis Inhibitory Factor") AND ("Interleukin-6" OR "Interleukin 6" OR "B-Cell Differentiation Factor" OR "B Cell Differentiation Factor" OR "B-Cell Differentiation Factor-2" OR "B Cell Differentiation Factor 2" OR "B-Cell Stimulatory Factor 2" OR "B-Cell Stimulatory Factor-2" OR "BSF-2" OR "Differentiation Factor, B-Cell" OR "Differentiation Factor, B Cell" OR "Differentiation Factor-2, B-Cell" OR "Differentiation Factor 2, B Cell" OR "Hepatocyte-Stimulating Factor" OR "Hepatocyte Stimulating Factor" OR "Hybridoma Growth Factor" OR "Growth Factor, Hybridoma" OR "IFN-beta 2" OR "IL-6" OR "IL6" OR "MGI-2" OR "Myeloid Differentiation-Inducing Protein" OR "Differentiation-Inducing Protein, Myeloid" OR "Myeloid Differentiation Inducing Protein" OR "Plasmacytoma Growth Factor" OR "Growth Factor, Plasmacytoma" OR "B Cell Stimulatory Factor-2" OR "B Cell Stimulatory Factor 2") AND ("STAT3 Transcription Factor" OR "Transcription Factor, STAT3" OR "APRF Transcription Factor" OR "Transcription Factor, APRF" OR "IL6-Response Factor" OR "IL6 Response Factor" OR "Signal Transducer and Activator of Transcription 3" OR "STAT3 Protein" OR "Acute-Phase Response Factor" OR "Acute Phase Response Factor" OR "Response Factor, Acute-Phase" OR "LIF-Response Factor" OR "LIF Response Factor" OR "STAT3b Transcription Factor" OR "Transcription Factor, STAT3b" OR "Stat3beta Transcription Factor" OR "Transcription Factor, Stat3beta" OR "STAT3a Transcription Factor" OR "Transcription Factor, STAT3a" OR "Stat3alpha Transcription Factor" OR "Transcription Factor, Stat3alpha" OR "STAT3" OR "STAT-3")

Search Fields: Title

This search resulted in 8 papers.

Reference 23 was part of this search.

1. ("NFKB" OR "NF-κB" OR "NF-kappa-B" OR "nuclear factor of kappa light polypeptide gene enhancer in B-cells") AND (inflammation)

Search fields: Title

This search resulted in 636 papers.

Reference 24 was part of this search.

1. ("Transforming Growth Factor" OR "Transforming Growth Factor beta" OR "TGFb" OR "Milk Growth Factor" OR "Factor, Milk Growth" OR "Growth Factor, Milk" OR "TGF-beta" OR "TGFbeta" OR "Platelet Transforming Growth Factor" OR "Bone-Derived Transforming Growth Factor" OR "Bone Derived Transforming Growth Factor") AND (rheumatoid OR rheumatic OR arthritis OR arthritides OR joint OR synovium OR synovial)

Search fields: Title

This search resulted in 161 papers.

Reference 26 was part of this search.

1. ("NFKB" OR "NF-κB" OR "NF-kappa-B" OR "nuclear factor of kappa light polypeptide gene enhancer in B-cells") AND (ubiquitination)

Search fields: Title

This search resulted in 79 papers.

Reference 27 was part of this search.

1. ("Tumor Necrosis Factor alpha" OR "Tumor Necrosis Factor-alpha" OR "Cachectin-Tumor Necrosis Factor" OR "Cachectin Tumor Necrosis Factor" OR "TNFalpha" OR "TNF-alpha" OR "Tumor Necrosis Factor" OR "Tumor Necrosis Factor Ligand Superfamily Member 2" OR "Cachectin" OR "TNF Superfamily, Member 2" OR “TNF”) AND ("I-kappa B Kinase" OR "B Kinase, I-kappa" OR "Kinase, I-kappa B" OR "IkappaB Kinase" OR "Kinase, IkappaB" OR "I kappa B Kinase" OR "IKK")

Search Fields: Title

This search resulted in 88 papers.

Reference 28 was part of this search.

1. ("TNF Receptor-Associated Factor 6" OR "TNF Receptor Associated Factor 6" OR "TRAF6" OR "TRAF-6 Protein" OR "TRAF 6 Protein") AND ("I-kappa B Kinase" OR "B Kinase, I-kappa" OR "Kinase, I-kappa B" OR "IkappaB Kinase" OR "Kinase, IkappaB" OR "I kappa B Kinase" OR "IKK")

Search Fields: Title

This search resulted in 15 papers.

References 29 and 31 were part of this search.

1. ("Interferon-alpha" OR "Receptor, Interferon alpha-beta" OR "interferon-gamma" OR "interferon-beta" OR "interferon gamma" OR "interferon alpha" OR "interferon beta" OR "IFN-gamma" OR "IFN-alpha" OR "IFN-beta" OR "IFN" OR "gamma-Interferon" OR "Interferon, Immune" OR "Immune Interferon" OR "Type II Interferon" OR "Interferon, Type II" OR "Interferon Type II" OR "Interferon, gamma" OR "Interferon-gamma" OR "Interferon-alpha" OR "Interferon alpha" OR "Interferon Alfa" OR "Interferon, Lymphoblastoid" OR "Lymphoblastoid Interferon" OR "Interferon, Leukocyte" OR "Leukocyte Interferon" OR "Interferon, Lymphoblast" OR "Lymphoblast Interferon" OR "alpha-Interferon" OR "alpha Interferon" OR "Interferon, alpha" OR "Interferon alpha-17" OR "Interferon alpha 17" OR "Interferon alpha-T" OR "Interferon alpha T" OR "LeIF I" OR "Interferon alpha-88" OR "Interferon alpha 88" OR "Interferon alpha-7" OR "Interferon alpha 7" OR "LeIF J" OR "Interferon alpha-J" OR "Interferon alpha J" OR "Interferon alpha-4" OR "Interferon alpha 4" OR "Interferon alpha4" OR "Interferon alpha-5" OR "Interferon alpha 5" OR "Interferon alpha5" OR "IFN-alpha5" OR "IFN alpha5" OR "Interferon alpha-1" OR "Interferon alpha 1" OR "Leif D" OR "D, Leif" OR "IFN-alpha D" OR "IFN alpha D" OR "Interferon alpha-2" OR "Interferon alpha 2" OR "LeIF A" OR "Interferon alpha-A" OR "Interferon alpha A" OR "Interferon-alpha 2" OR "2, Interferon-alpha" OR "IFN-alpha 2" OR "IFN-alpha-2" OR "Interferon-beta" OR "Interferon beta" OR "Interferon, Fibroblast" OR "Fibroblast Interferon" OR "beta-Interferon" OR "beta Interferon" OR "Fiblaferon" OR "Biosyn Brand of Interferon-beta" OR "Biosyn Brand of Interferon beta" OR "Interferon-beta Biosyn Brand" OR "beta-1 Interferon" OR "beta 1 interferon" OR "Interferon-beta1" OR "Interferon beta1" OR "Interferon, beta-1" OR "Interferon, beta 1") AND ("STAT1 Transcription Factor" OR "Transcription Factor, STAT1" OR "Transcription Factor STAT91" OR "STAT-91 Transcription Factor" OR "STAT 91 Transcription Factor" OR "Transcription Factor, STAT-91" OR "Signal Transducer and Activator of Transcription 1" OR "STAT-91 Protein" OR "STAT 91 Protein" OR "STAT91 Transcription Factor" OR "Transcription Factor, STAT91" OR "STAT1 Protein" OR "Gamma-Activated Factor, 91 kDa" OR "Gamma Activated Factor, 91 kDa" OR "STAT1" OR "STAT-1")

Search Fields: Title

This search resulted in 399 papers.

References 30, 42, 43, 45, 60 were part of this search.

1. ("STAT1 Transcription Factor" OR "Transcription Factor, STAT1" OR "Transcription Factor STAT91" OR "STAT-91 Transcription Factor" OR "STAT 91 Transcription Factor" OR "Transcription Factor, STAT-91" OR "Signal Transducer and Activator of Transcription 1" OR "STAT-91 Protein" OR "STAT 91 Protein" OR "STAT91 Transcription Factor" OR "Transcription Factor, STAT91" OR "STAT1 Protein" OR "Gamma-Activated Factor, 91 kDa" OR "Gamma Activated Factor, 91 kDa" OR "STAT1" OR "STAT-1") AND ("NOS2" OR "iNOS" OR "inducible nitric oxide synthase" OR "HEP-NOS" OR "NOS2A" OR "NOS-2" OR "Nanos2" OR "Nanos homolog 2" OR "inducible nitric oxide 2A" OR "Nitric Oxide Synthase Type II" OR "Inducible NOS Protein" OR "INOS Enzyme" OR "Nitric Oxide Synthase, Type II" OR "NOS-II" OR "NOS II" OR "Inducible Nitric Oxide Synthase" OR "Nitric Oxide Synthase II" OR "nitric oxide synthase-2” OR “inducible NO synthase”)

Search fields: Title

This search resulted in 29 papers.

References 33, 42, 45 were part of this search.

Also references 19 and 32 in the S2 signalling_network were part of this search.

1. ("CCAAT-Enhancer-Binding Protein-beta" OR "CCAAT Enhancer Binding Protein beta" OR "Protein-beta, CCAAT-Enhancer-Binding" OR "AGP-EBP Transcription Factor" OR "AGP EBP Transcription Factor" OR "Transcription Factor, AGP-EBP" OR "C-EBP beta" OR "C EBP beta" OR "C-EBP-beta" OR "C-EBP-Related Protein 2" OR "2, C-EBP-Related Protein" OR "C EBP Related Protein 2" OR "NF-IL6" OR "CRP2 Protein" OR "IL-6 DBP" OR "IL-6-Dependent DNA Binding Protein" OR "IL 6 Dependent DNA Binding Protein" OR "Interleukin-6 Nuclear Factor" OR "Interleukin 6 Nuclear Factor" OR "Nuclear Factor, Interleukin-6" OR "LAP Transcription Factor" OR "Transcription Factor, LAP" OR "40-C-EBP Protein" OR "C-EBPbeta" OR "C EBPbeta" OR "Liver-Enriched Inhibiting Protein" OR "Inhibiting Protein, Liver-Enriched" OR "Liver Enriched Inhibiting Protein" OR "Liver-Enriched Inhibitory Protein, LIP" OR "Liver Enriched Inhibitory Protein, LIP" OR "23-C-EBP Protein") AND ("NOS2" OR "iNOS" OR "inducible nitric oxide synthase" OR "HEP-NOS" OR "NOS2A" OR "NOS-2" OR "Nanos2" OR "Nanos homolog 2" OR "inducible nitric oxide 2A" OR "Nitric Oxide Synthase Type II" OR "Inducible NOS Protein" OR "INOS Enzyme" OR "Nitric Oxide Synthase, Type II" OR "NOS-II" OR "NOS II" OR "Inducible Nitric Oxide Synthase" OR "Nitric Oxide Synthase II" OR "nitric oxide synthase-2” OR “inducible NO synthase”)

This search resulted in 93 papers.

Reference 35 was part of this search.

1. ("Interferon-alpha" OR "Receptor, Interferon alpha-beta" OR "interferon-gamma" OR "interferon-beta" OR "interferon gamma" OR "interferon alpha" OR "interferon beta" OR "IFN-gamma" OR "IFN-alpha" OR "IFN-beta" OR "IFN" OR "gamma-Interferon" OR "Interferon, Immune" OR "Immune Interferon" OR "Type II Interferon" OR "Interferon, Type II" OR "Interferon Type II" OR "Interferon, gamma" OR "Interferon-gamma" OR "Interferon-alpha" OR "Interferon alpha" OR "Interferon Alfa" OR "Interferon, Lymphoblastoid" OR "Lymphoblastoid Interferon" OR "Interferon, Leukocyte" OR "Leukocyte Interferon" OR "Interferon, Lymphoblast" OR "Lymphoblast Interferon" OR "alpha-Interferon" OR "alpha Interferon" OR "Interferon, alpha" OR "Interferon alpha-17" OR "Interferon alpha 17" OR "Interferon alpha-T" OR "Interferon alpha T" OR "LeIF I" OR "Interferon alpha-88" OR "Interferon alpha 88" OR "Interferon alpha-7" OR "Interferon alpha 7" OR "LeIF J" OR "Interferon alpha-J" OR "Interferon alpha J" OR "Interferon alpha-4" OR "Interferon alpha 4" OR "Interferon alpha4" OR "Interferon alpha-5" OR "Interferon alpha 5" OR "Interferon alpha5" OR "IFN-alpha5" OR "IFN alpha5" OR "Interferon alpha-1" OR "Interferon alpha 1" OR "Leif D" OR "D, Leif" OR "IFN-alpha D" OR "IFN alpha D" OR "Interferon alpha-2" OR "Interferon alpha 2" OR "LeIF A" OR "Interferon alpha-A" OR "Interferon alpha A" OR "Interferon-alpha 2" OR "2, Interferon-alpha" OR "IFN-alpha 2" OR "IFN-alpha-2" OR "Interferon-beta" OR "Interferon beta" OR "Interferon, Fibroblast" OR "Fibroblast Interferon" OR "beta-Interferon" OR "beta Interferon" OR "Fiblaferon" OR "Biosyn Brand of Interferon-beta" OR "Biosyn Brand of Interferon beta" OR "Interferon-beta Biosyn Brand" OR "beta-1 Interferon" OR "beta 1 interferon" OR "Interferon-beta1" OR "Interferon beta1" OR "Interferon, beta-1" OR "Interferon, beta 1") AND ("NOS2" OR "iNOS" OR "inducible nitric oxide synthase" OR "HEP-NOS" OR "NOS2A" OR "NOS-2" OR "Nanos2" OR "Nanos homolog 2" OR "inducible nitric oxide 2A" OR "Nitric Oxide Synthase Type II" OR "Inducible NOS Protein" OR "INOS Enzyme" OR "Nitric Oxide Synthase, Type II" OR "NOS-II" OR "NOS II" OR "Inducible Nitric Oxide Synthase" OR "Nitric Oxide Synthase II" OR "nitric oxide synthase-2” OR “inducible NO synthase”)

Search Fields: Title

This search resulted in 189 papers.

References 36, 42, 45, 46, 50 were part of this search.

1. ("STAT1 Transcription Factor" OR "Transcription Factor, STAT1" OR "Transcription Factor STAT91" OR "STAT-91 Transcription Factor" OR "STAT 91 Transcription Factor" OR "Transcription Factor, STAT-91" OR "Signal Transducer and Activator of Transcription 1" OR "STAT-91 Protein" OR "STAT 91 Protein" OR "STAT91 Transcription Factor" OR "Transcription Factor, STAT91" OR "STAT1 Protein" OR "Gamma-Activated Factor, 91 kDa" OR "Gamma Activated Factor, 91 kDa" OR "STAT1" OR "STAT-1") AND (“phosphorylation”)

Search fields: Title

This search resulted in 153 papers.

Reference 39 and 43 was part of this search.

1. ("Receptors, Interleukin-" OR "Interleukin 1 Receptor Antagonist Protein" OR "IL1" OR "Interleukin 1" OR "IL-1") AND ("STAT1 Transcription Factor" OR "Transcription Factor, STAT1" OR "Transcription Factor STAT91" OR "STAT-91 Transcription Factor" OR "STAT 91 Transcription Factor" OR "Transcription Factor, STAT-91" OR "Signal Transducer and Activator of Transcription 1" OR "STAT-91 Protein" OR "STAT 91 Protein" OR "STAT91 Transcription Factor" OR "Transcription Factor, STAT91" OR "STAT1 Protein" OR "Gamma-Activated Factor, 91 kDa" OR "Gamma Activated Factor, 91 kDa" OR "STAT1" OR "STAT-1")

Search fields: Title

This search resulted in 5 papers.

Reference 40 was part of this search.

1. ("STAT1 Transcription Factor" OR "Transcription Factor, STAT1" OR "Transcription Factor STAT91" OR "STAT-91 Transcription Factor" OR "STAT 91 Transcription Factor" OR "Transcription Factor, STAT-91" OR "Signal Transducer and Activator of Transcription 1" OR "STAT-91 Protein" OR "STAT 91 Protein" OR "STAT91 Transcription Factor" OR "Transcription Factor, STAT91" OR "STAT1 Protein" OR "Gamma-Activated Factor, 91 kDa" OR "Gamma Activated Factor, 91 kDa" OR "STAT1" OR "STAT-1") AND (“inflammation” OR “inflammatory”)

Search fields: Title

This search resulted in 74 papers.

Reference 41 was part of this search.

1. ("STAT3 Transcription Factor" OR "Transcription Factor, STAT3" OR "APRF Transcription Factor" OR "Transcription Factor, APRF" OR "IL6-Response Factor" OR "IL6 Response Factor" OR "Signal Transducer and Activator of Transcription 3" OR "STAT3 Protein" OR "Acute-Phase Response Factor" OR "Acute Phase Response Factor" OR "Response Factor, Acute-Phase" OR "LIF-Response Factor" OR "LIF Response Factor" OR "STAT3b Transcription Factor" OR "Transcription Factor, STAT3b" OR "Stat3beta Transcription Factor" OR "Transcription Factor, Stat3beta" OR "STAT3a Transcription Factor" OR "Transcription Factor, STAT3a" OR "Stat3alpha Transcription Factor" OR "Transcription Factor, Stat3alpha" OR "STAT3" OR "STAT-3") AND ("NOS2" OR "iNOS" OR "inducible nitric oxide synthase" OR "HEP-NOS" OR "NOS2A" OR "NOS-2" OR "Nanos2" OR "Nanos homolog 2" OR "inducible nitric oxide 2A" OR "Nitric Oxide Synthase Type II" OR "Inducible NOS Protein" OR "INOS Enzyme" OR "Nitric Oxide Synthase, Type II" OR "NOS-II" OR "NOS II" OR "Inducible Nitric Oxide Synthase" OR "Nitric Oxide Synthase II" OR "nitric oxide synthase-2” OR “inducible NO synthase”)

Search fields: Title

This search resulted in 45 papers.

References 42, 47, 48 was part of this search.

1. ("Interleukin-4" OR "Interleukin 4" OR "B-Cell Growth Factor-1" OR "B Cell Growth Factor 1" OR "B-Cell Growth Factor-I" OR "B Cell Growth Factor I" OR "B-Cell Proliferating Factor" OR "B Cell Proliferating Factor" OR "B-Cell Stimulating Factor-1" OR "B Cell Stimulating Factor 1" OR "B-Cell Stimulatory Factor 1" OR "B-Cell Stimulatory Factor-1" OR "BCGF-1" OR "Binetrakin" OR "BSF-1" OR "IL-4" OR "IL4" OR "Mast Cell Growth Factor-2" OR "Mast Cell Growth Factor 2" OR "MCGF-2" OR "B Cell Stimulatory Factor-1" OR "B Cell Stimulatory Factor 1") AND ("NOS2" OR "iNOS" OR "inducible nitric oxide synthase" OR "HEP-NOS" OR "NOS2A" OR "NOS-2" OR "Nanos2" OR "Nanos homolog 2" OR "inducible nitric oxide 2A" OR "Nitric Oxide Synthase Type II" OR "Inducible NOS Protein" OR "INOS Enzyme" OR "Nitric Oxide Synthase, Type II" OR "NOS-II" OR "NOS II" OR "Inducible Nitric Oxide Synthase" OR "Nitric Oxide Synthase II" OR "nitric oxide synthase-2” OR “inducible NO synthase”)

Search fields: Title

This search resulted in 19 papers.

Reference 49 was part of this search.

1. ("Interleukin-4" OR "Interleukin 4" OR "B-Cell Growth Factor-1" OR "B Cell Growth Factor 1" OR "B-Cell Growth Factor-I" OR "B Cell Growth Factor I" OR "B-Cell Proliferating Factor" OR "B Cell Proliferating Factor" OR "B-Cell Stimulating Factor-1" OR "B Cell Stimulating Factor 1" OR "B-Cell Stimulatory Factor 1" OR "B-Cell Stimulatory Factor-1" OR "BCGF-1" OR "Binetrakin" OR "BSF-1" OR "IL-4" OR "IL4" OR "Mast Cell Growth Factor-2" OR "Mast Cell Growth Factor 2" OR "MCGF-2" OR "B Cell Stimulatory Factor-1" OR "B Cell Stimulatory Factor 1") AND ("STAT6 Transcription Factor" OR "Transcription Factor, STAT6" OR "STAT6 Protein" OR "Signal Transducer and Activator of Transcription 6" OR "STAT6" OR "STAT-6" OR "STAT")

Search Fields: Title

This search resulted in 234 papers.

Reference 28 and 29 in S2 signalling_network were part of this search.

1. ("STAT1 Transcription Factor" OR "Transcription Factor, STAT1" OR "Transcription Factor STAT91" OR "STAT-91 Transcription Factor" OR "STAT 91 Transcription Factor" OR "Transcription Factor, STAT-91" OR "Signal Transducer and Activator of Transcription 1" OR "STAT-91 Protein" OR "STAT 91 Protein" OR "STAT91 Transcription Factor" OR "Transcription Factor, STAT91" OR "STAT1 Protein" OR "Gamma-Activated Factor, 91 kDa" OR "Gamma Activated Factor, 91 kDa" OR "STAT1" OR "STAT-1") AND (rheumatoid OR rheumatic OR arthritis OR arthritides OR disease OR diseases)

Search fields: Title

This search resulted in 49 papers.

Reference 56 was part of this search.

1. ("NOS2" OR "iNOS" OR "inducible nitric oxide synthase" OR "HEP-NOS" OR "NOS2A" OR "NOS-2" OR "Nanos2" OR "Nanos homolog 2" OR "inducible nitric oxide 2A" OR "Nitric Oxide Synthase Type II" OR "Inducible NOS Protein" OR "INOS Enzyme" OR "Nitric Oxide Synthase, Type II" OR "NOS-II" OR "NOS II" OR "Inducible Nitric Oxide Synthase" OR "Nitric Oxide Synthase II" OR "nitric oxide synthase-2” OR “inducible NO synthase”) AND (“rac”)

Search fields: Title

This search resulted in 1 paper.

Reference 63 was part of this search.

1. Reference 17 was searched in Google Scholar using the title of the article.

To select the references from the search hits, we have read the abstracts of the papers.

1. The search terms for the references [13, 16, 19-50] used to construct the network are included in the list above.
