# Supplementary material for: A Cytokine Signalling Network for the Regulation of Inducible Nitric Oxide Synthase Expression in Rheumatoid Arthritis
Source: PLoS One. 2016 Sep 14;11(9):e0161306. doi: 10.1371/journal.pone.0161306 (PMC5023176; doi:10.1371/journal.pone.0161306)
Supplement: S2 File — (DOCX) [file pone.0161306.s009.docx]

**Description of the cytokine signalling network regulating iNOS expression**

In this study, the signalling pathways of the cytokines leading to the regulation of the transcription factors or modulator proteins associated with iNOS expression are consolidated into a network (S1 Fig). The cytokines TNF-α, IL-1β, IFN-γ, IL-10, IL-4, IL-6 and TGF-β are involved in the activation of transcription factors such as NF-κB, STAT1, IRF1, STAT3, STAT6, Oct-1, C/EBPβ and AP-1 [1, 2-4, 5]. The promoter region of human iNOS shows multiple binding sites for these transcription factors [6, 7, 8]. The details of the network are described below.

**S1 Fig. The cytokine signalling network regulating expression of iNOS.** The network has two compartments; cytosol and nucleus. The nucleus is represented by the green-bordered box while the remaining network is within the yellow-bordered box representing cytosol. The yellow border represents the cell membrane. The network is constructed in CellDesigner and the following symbols therein are adapted: the light green rectangles are proteins, the green trapeziums are mRNAs, the yellow rectangles are genes, the pale yellow coloured structures on the cell membrane are receptors and the boxes with stacked proteins are the complexes. The purple coloured box represents the balance between GTP-bound and GDP-bound RAC2. The blue lines ending with circles represent activation, brown lines ending with circles represent ubiquitination and the red lines represent inhibition. The black arrows denote state transition, the orange arrows denote transportation across compartments, the green arrows denote transcription and the pink arrows denote translation.

**Activation of NF-κB by TNF-α, IL-1β, IFN-γ and TGF-β**

The signalling network of TNF-α, IL-1β, IFN-γ and TGF-β that leads to the activation of NF-κB is extracted from S1 Figand is shown in S2 Fig. NF-κB usually exists in an inactive state as a complex with any of the nuclear factor of kappa light polypeptide gene enhancer in B-cells inhibitor, alpha, beta or epsilon (IκBα, β or ε). TNF-α, on binding to its receptor, stimulates formation of K63-linked ubiquitinated receptor interacting protein 1 (RIP-1) via TNF receptor associated factor 2 (TRAF2) [9]. Ubiquitinated RIP-1 facilitates recruitment of transforming growth factor beta-activated kinase 1 (TAK1) complex (TAK1-binding protein 1 (TAB1), TAB2, TAB3 and TAK1) and IκB kinase (IKK) complex (IKK-α, IKK-β and NEMO) to the TNF receptor complex [10]. Subsequently, TAK1 and IKK are activated. Another pro-inflammatory cytokine, IL1β, on binding to its receptor, stimulates formation of K63-linked ubiquitinated interleukin-1 receptor-associated kinase 1 (IRAK1) via TRAF6 [11]. This is followed by the activation of TAK1 and IKK in the cytoplasm [10]. The pro-inflammatory cytokine, IFN-γ induces phosphorylation of p38 mitogen-activated protein kinase (p38 MAPK) through JAK pathway. The activated p38 MAPK is involved in IKK activation [12]. IKK catalyses the degradation of IκBα and consequently activates NF-κB. Moreover, TNF-α stimulates activation of the MKK4 - MEKK1 - JNK pathway which enhances iNOS expression possibly via activation of NF-κB [13]. IFN-γ, also via a JAK-dependent and STAT1-independent mechanism, activates protein kinase R (PKR) [14]. Subsequently, the active PKR degrades IκBβ to release an active NF-κB [14]. The canonical signalling pathway of TGF-β, an anti-inflammatory cytokine, involves the activation of Smad proteins [15]. However, TGF-β on binding to its receptor, alternatively stimulates K63-linked polyubiquitination of TAK1 via TRAF6 [5]. The activated TAK1, in turn, activates IKK/ NF-κB. The activated NF-κB relocates to the nucleus and induces the transcription of NFκB-responsive genes like iNOS. NF-κB also induces the expression of its feedback regulator A20 which is a deubiquitination enzyme. A20 inhibits IL-1β and TNF-α stimulated IKK/ NF-κB activation by preventing TRAF6 mediated K63-ubiquitination and targeting RIP-1 proteasomal degradation respectively. The Cylindromatosis (Turban Tumor Syndrome) (CYLD) inhibits the signalling of TNF-α and IL-1β by cleaving K63 linked polyubiquitin chain on RIP-1 and NEMO respectively.

**S2 Fig. The activation of NF-κB by TNF-α, IL-1β, IFN-γ and TGF-β.** The network has two compartments; cytosol and nucleus. The nucleus is represented by the green-bordered box while the remaining network is within the yellow-bordered box representing cytosol. The yellow border represents the cell membrane. The network is constructed in CellDesigner and the following symbols therein are adapted: the light green rectangles are proteins, the green trapeziums are mRNAs, the yellow rectangles are genes, the pale yellow coloured structures on the cell membrane are receptors and the boxes with stacked proteins are the complexes. The blue lines ending with circles represent activation, brown lines ending with circles represent ubiquitination whereas the red lines represent inhibition. The black arrows denote state transition, the orange arrows denote transportation across compartments, the green arrows denote transcription and the pink arrows denote translation.

**Regulation of STAT1 phosphorylation at Y701 by TGF-β and the intermediate kinases of IFN**

The signalling network of STAT1 activation by intermediate kinases of IFN and STAT1 inhibition by TGF-β is extracted from S1 Figand is shown in S3 Fig. IFN-γ, a type II interferon, is the prime activator of STAT1. On binding to the ligand, the IFN-γ receptors phosphorylate the receptor-bound janus kinase 1 (JAK1) and kinase 2 (JAK2). The phosphorylated JAKs in turn phosphorylate the IFN-γ receptor. Thus, the receptor forms a docking site enabling the binding of STAT1 to it. The JAKs phosphorylate the tyrosine residue Y701 of the bound STAT1 [16]. The phosphorylation of Y701 of STAT1 is intensified by p38MAPK which is activated by IFN-γ [12]. The phosphorylation of STAT1 enables the formation of STAT1 homo-dimer which later gets translocated into the nucleus. The STAT1 homo-dimer binds to the γ-activated site (GAS) of the promoter and turns on the IFN-responsive genes like iNOS [4].

**S3 Fig. The regulation of STAT1 phosphorylation at Y701 by TGF-β and the intermediate kinases of IFN.** The network has two compartments; cytosol and nucleus. The nucleus is represented by the green-bordered box while the remaining network is within the yellow-bordered box representing cytosol. The yellow border represents the cell membrane. The network is constructed in CellDesigner and the following symbols therein are adapted: the light green rectangles are proteins, the green trapeziums are mRNAs, the yellow rectangles are genes, the pale yellow coloured structures on the cell membrane are receptors and the boxes with stacked proteins are the complexes. The blue lines ending with circles represent activation and the red lines represent inhibition. The black arrows denote state transition, the orange arrows denote transportation across compartments, the green arrows denote transcription and the pink arrows denote translation.

STAT1 is also activated by type I interferons IFN-α and IFN-β. The type I interferon receptors facilitate phosphorylation of JAK1 and tyrosine kinase 2 (TYK2). The JAKs catalyse the phosphorylation of the residues Y660 of STAT2 and Y701 of STAT1 [16]. The phosphorylated STAT1 and STAT2 form a heterodimer, STAT1:STAT2, which can go on to form a complex with a third transcription factor interferon regulatory factor 9 (IRF9). This tri-molecular complex is known as IFN-stimulated gene factor 3 (ISGF3). ISGF3 can thereafter migrate to the nucleus and bind to the interferon stimulated response elements (ISRE) site of IFNα/β inducible genes. The activated heterodimer of STAT1 and STAT2 can also move into the nucleus and bind to the GAS site to trigger the transcription of IFN-responsive genes like IRF1 [16]. The formation of the complex consisting of IRF1 and IFN consensus sequence binding protein (ICSBP) precedes the binding of the complex to the ISRE site in the iNOS promoter to induce IFN-γ mediated iNOS expression [17]. Interestingly, IL-4 prevents the formation of this complex to suppress IFN-γ stimulated expression of iNOS [17].

Chesrown et al, from experiments on murine cells, have demonstrated that TGF-β suppresses IFN-γ induced iNOS expression [18]. TGF-β, on binding, activates its receptor TGF-βR1 which induces phosphorylation of IFNGR1, the receptor of IFN-γ [19]. This prevents binding of STAT1 to IFNGR1 and therefore inhibits activation of STAT1. Thus TGF-β inhibits IFN-γ mediated STAT1 activation to supress iNOS expression.

**Activation of STAT1 phosphorylation at S727 by IFN-γ, IL-1β and TNF-α**

The signalling network of STAT1 activation due to its phosphorylation at serine 727 by IL-1β, TNF-α and IFN-γ is extracted from S1 Fig and is shown in S4 Fig. It is evident from experimental studies that STAT1 phosphorylated at serine 727 binds to IFN-γ activated site (GAS) on iNOS promoter to promote its transcription [20]. IFN-γ signals phosphorylation of STAT1 at serine 727 by a JAK2-dependent mechanism [21]. Experimental studies also elicited an IL-1β-stimulated activation of STAT1 phosphorylation at serine 727. STAT1 interacts with IRAK and TRAF6 prior to its phosphorylation at the serine residue [22, 23]. Lee et al demonstrated that IL-1β induces STAT1 phosphorylation at the serine residue in a p38 MAPK dependent manner [24]. The serine phosphorylated STAT1 induces transcription of IRF1 which is an essential transcription factor of iNOS [24]. In addition to IL-1β, the pro-inflammatory cytokine TNF-α also phosphorylates STAT1 at the serine residue by a p38 MAPK dependent mechanism [25]. Moreover, it is observed that IL-1β mediated phosphorylation of serine 727 of STAT1 is potentiated by the canonical phosphorylation of STAT1 at its tyrosine residue by IFN-γ [20]. Therefore, in addition to the tyrosine 701 phosphorylation of STAT1, serine 727 phosphorylation of STAT1 stimulated by the alternative signalling pathways of IFN-γ, IL-1β and TNF-α is required for the augmentation of iNOS transcription.

**S4 Fig. The activation of STAT1 phosphorylation at S727 by IFN-γ, IL-1β and TNF-α.** The network has two compartments; cytosol and nucleus. The nucleus is represented by the green-bordered box while the remaining network is within the yellow-bordered box representing cytosol. The yellow border represents the cell membrane. The network is constructed in CellDesigner and the following symbols therein are adapted: the light green rectangles are proteins, the green trapeziums are mRNAs, the yellow rectangles are genes, the pale yellow coloured structures on the cell membrane are receptors and the boxes with stacked proteins are the complexes. The blue lines ending with circles represent activation and the brown lines ending with circles represent ubiquitination. The black arrows denote state transition, the orange arrows denote transportation across compartments, the green arrows denote transcription and the pink arrows denote translation.

**Activation of STAT1, STAT3 and STAT6 by IL-10, 1L-6 and IL-4**

The signalling network of STAT3, STAT1 and STAT6 activation by IL-10, IL-6 and IL-4 is extracted from S1 Fig and is shown in S5 Fig. IL-10 receptors IL10R1 and IL10R2 – upon binding to IL-10 – facilitate the activation of JAK1 and TYK2. The activated JAKs aid in creating a docking site for STAT3 and STAT1 on IL-10 receptors. Subsequently, JAKs activate STAT3 and STAT1. The IL-6 receptor (gp130), on binding to IL-6, activates JAK1, JAK2 and TYK2. The JAKs promote activation of STAT3 which relocates to the nucleus to turn on STAT3-responsive genes like suppressor of cytokine signalling 3 (SOCS3).

**S5 Fig. The activation of STAT1, STAT3 and STAT6 by IL-10, 1L-6 and IL-4.** The network has two compartments; cytosol and nucleus. The nucleus is represented by the green-bordered box while the remaining network is within the yellow-bordered box representing cytosol. The yellow border represents the cell membrane. The network is constructed in CellDesigner and the following symbols therein are adapted: the light green rectangles are proteins, the green trapeziums are mRNAs, the yellow rectangles are genes, the pale yellow coloured structures on the cell membrane are receptors and the boxes with stacked proteins are the complexes. The blue lines ending with circles represent activation and the red lines represent inhibition. The black arrows denote state transition, the orange arrows denote transportation across compartments, the green arrows denote transcription and the pink arrows denote translation.

The activated STAT3 after being translocated to the nucleus, interacts with the epidermal growth factor receptor (EGFR) to form a complex. This STAT3/ EGFR complex, upon binding to iNOS promoter, enhances iNOS transcription [26]. In contrast, the activated STAT3 binds to the DNA binding domain of p65 subunit and to the trans-activator p300/CBP of NF-κB. Thus STAT3 suppresses iNOS expression by restricting the binding of NF-κB to the iNOS promoter and also by competing with the trans-activator binding to NF-κB [27].

The two prominent feedback inhibitory loops in the network are initiated after the induction of SOCS3 and SOCS1 by IL-6 and IL-10. The expression of SOCS1 and SOCS3 plays a role in determining the balance between the activation of STAT3 and STAT1 which governs the expression of iNOS. The cytokine IL-6 is a strong inducer for SOCS3 and a weak inducer for SOCS1. The phosphorylated residue, Y759, of the IL-6 gp130 receptor is responsible for the recruitment of SOCS3 [2]. SOCS3 binds to the IL-6 receptor and negatively regulates IL-6-mediated JAK and STAT3 phosphorylation. IL-10 is a potent inducer of SOCS-1 and SOCS-3 but its receptor lacks a motif to which SOCS1 and SOCS3 can bind. It is noted that IL-10 signalling is weakly inhibited by SOCS3 but strongly inhibited by SOCS1 [2]. The ability of SOCS1 to dephosphorylate JAKs might be the key mechanism in the attenuation of IL-10 signalling.

The anti-inflammatory cytokine IL-4 predominantly activates the transcription factor STAT6. Activation of the IL-4-STAT6 pathway is confirmed in the affected RA synovium [28]. The IL-4 receptor, upon binding to its ligand, provokes phosphorylation of JAKs which in turn catalyse the phosphorylation of STAT6 attached to the IL-4 receptor. The activated STAT6 forms a homodimer which then enters the nucleus. STAT6 represses transcription of IRF1 and also inhibits NF-κB driven transcriptions [29]. Thus, STAT6 is involved in the negative regulation of iNOS.

Among the protein tyrosine phosphatases, Src homology region 2 domain-containing phosphatase-1 (SHP-1) and phosphatase-2 (SHP-2) are involved in dephosphorylating JAKs and STATs. SHP-1 inhibits STATs via the de-phosphorylation of JAK2 [30]. SHP-2 dephosphorylates Y701 of STAT-1 [30]. SHP-2 attaches to the IL-6 gp130 receptor and negatively regulates the phosphorylation of STAT3 [2]. Thus, SHP-2 efficiently weakens IL-6 signalling. It is experimentally observed that both IL-6 and IL-10 induce activation of SHP-2 in murine macrophages [2]. The mechanism by which SHP-2 influences IL-10 signalling is not very clear.

**Activation of Smad proteins by TGF-β**

The signalling network for iNOS suppression mediated by TGF-β activated Smads is extracted from S1 Fig and is shown in S6 Fig. The cytokine TGF-β down-regulates the expression of iNOS and thereby NO in synoviocytes derived from human RA patients [31]. Earlier experiments on mouse suggest that TGF-β inhibits iNOS expression via the transcriptional modulator proteins Smad2 and Smad3 [32]. TGF-β is released from a complex containing latent TGF-β binding proteins by the action of Thrombospondin 1 [33]. The TGF-β receptor, on binding to its ligand, recruits the Smad proteins near the cell membrane. Subsequently, the Smads are phosphorylated. Among the Smads, Smad2 and Smad3 bind with Smad4 and move to the nucleus to regulate gene expression [15]. Inside the nucleus, Smad3 is known to sequester p300/CREB binding protein (p300/CREBBP) resulting in the inhibition of NF-κB target genes like iNOS [15]. TGF-β is also known to suppress iNOS expression by a Smad6-dependent mechanism. By the Smad6 and protein kinase C (PKC)-dependent pathway, TGF-β enhances the binding of a heterodimer TCF11/MafG to iNOS promoter. This leads to iNOS suppression [34].

**S6 Fig. The activation of Smad proteins by TGF**-**β.** The network has two compartments; cytosol and nucleus. The nucleus is represented by the green-bordered box while the remaining network is within the yellow-bordered box representing cytosol. The yellow border represents the cell membrane. The network is constructed in CellDesigner and the following symbols therein are adapted: the light green rectangles are proteins, the green trapeziums are mRNAs, the yellow rectangles are genes, the pale yellow coloured structures on the cell membrane are receptors and the boxes with stacked proteins are the complexes. The blue lines ending with circles represent activation and the red lines represent inhibition. The black arrows denote state transition, the orange arrows denote transportation across compartments, the green arrows denote transcription and the pink arrows denote translation.

**Activation of p300, C/EBPβ, AP-1 and Oct-1 by IFN-γ, TNF-α and IL1-β**

The signalling network describing iNOS transcription regulated by cytokine-induced activators like p300, C/EBPβ, AP-1 and Oct-1 is extracted from S1 Fig and is shown in S7 Fig. The transcription factor AP-1, a heterodimer of Fos (Fra2, cFos) and Jun (cJun, JunD) proteins, facilitates IFN-γ mediated iNOS transcription [35]. Experimental studies have demonstrated that p300, a transcriptional co-activator of iNOS, bridges AP-1 to the core promoter of iNOS in order to form a DNA loop. This DNA loop is necessary for iNOS transcription [8]. IFN-γ stimulates binding of p300 to NF-κB and IRF1. p300 acetylates the p50 subunit of NF-κB and thereby increases the binding of NF-κB to iNOS promoter. Thus, p300 is proven to be an essential transcriptional co-activator of iNOS upon cytokine stimulation of cells. IFN-γ stimulates the formation of phosphorylated STAT1 which interacts with c-Fos of AP-1 transcriptional complex. Subsequently, STAT1/c-Fos complex binds to the GAS site of iNOS promoter to induce transcription [35]. CCAAT/enhancer-binding protein-β (C/EBPβ) is an essential transcription factor for cytokine mediated expression of iNOS. Experimental studies on murine mesenchymal stem cells have confirmed that TNF-α and IFN-γ synergistically induce the expression of C/EBPβ which in turn binds to iNOS promoter to enhance iNOS expression [36]. It is also shown that TNF-α and IFN-γ induced C/EBPβ expression follows a STAT1-independent pathway [36]. It is also demonstrated that IL1-β enhances iNOS expression via C/EBPβ binding to iNOS promoter [37]. Another protein, octamer binding transcription factor 1 (Oct-1) belonging to the Pit-Oct-Unc (POU) domain family of transcription factors, binds to iNOS promoter to facilitate the transcription of iNOS when the cells are stimulated with a combination of cytokines like TNF-α, IL-1β and IFN-γ. However, neither of the extent of the binding of Oct-1 to iNOS promoter nor the transcriptional activity of Oct-1is influenced by the cytokines [38]. The activated STAT6, stimulated by IL-4, interacts with CREB-binding protein (CBP) which is a coactivator of Oct-1. Thus STAT6 competes with Oct-1 coactivator to mediate a transcriptional repression of iNOS [39].

**S7 Fig. The activation of p300, C/EBPβ, AP-1 and Oct-1 by IFN-γ, TNF-α and IL1-β.** The network has two compartments; cytosol and nucleus. The nucleus is represented by the green-bordered box while the remaining network is within the yellow-bordered box representing cytosol. The yellow border represents the cell membrane. The network is constructed in CellDesigner and the following symbols therein are adapted: the light green rectangles are proteins, the green trapeziums are mRNAs, the yellow rectangles are genes, the pale yellow coloured structures on the cell membrane are receptors and the boxes with stacked proteins are the complexes. The blue lines ending with circles represent activation. The black arrows denote state transition, the orange arrows denote transportation across compartments, the green arrows denote transcription and the pink arrows denote translation.

**References**

1. Coccia EM, Stellacci E, Marziali G, Weiss G, Battistini A. IFN-γ and IL-4 differently regulate inducible NO synthase gene expression through IRF-1 modulation. Int Immunol. 2000; 12(7): 977-985.
2. Niemand C, Nimmesgern A, Haan S, Fischer P, Schaper F, Rossaint R et al. Activation of STAT3 by IL-6 and IL-10 in primary human macrophages is differentially modulated by suppressor of cytokine signalling 3. J Immunol. 2003; 170: 3263-3272.
3. Lawrence T. The nuclear factor NF-κB pathway in inflammation. Cold Spring Harb Perspect Biol. 2009; 1(6):a001651.
4. Kleinert H, Schwarz PM, Förstermann U. Regulation of the expression of inducible nitric oxide synthase. Biol. Chem. 2003; 384(10-11), 1343-1364.
5. Mao R, Fan Y, Mou Y, Zhang H, Fu S, Yang J. TAK1 lysine 158 is required for TGF-β-induced TRAF6-mediated Smad-independent IKK/NF-κB and JNK/AP-1 activation. Cell Signal. 2011 January; 23(1): 222-227.
6. Aktan F. iNOS-mediated nitric oxide production and its regulation. Life Sci. 2004; 75(6):639-653.
7. Kröncke KD, Fehsel K, Kolb-Bachofen V. Inducible nitric oxide synthase in human diseases. Clin Exp Immunol. 1998; 113(2):147-156.
8. Guo Z, Zheng L, Liao X, Geller D. Up-regulation of human inducible nitric oxide synthase by p300 transcriptional complex. Plos ONE. 2016. 11(1): e0146640.
9. Habelhah H. Emerging complexity of protein ubiquitination in the NF-κB pathway. Genes & Cancer 2010; 1(7): 735-747.
10. Tarantino N, Tinevez J-Y, Crowell EF, Boisson B, Henriques R, Mhlanga M, et al. TNF and IL-1 exhibit distinct ubiquitin requirements for inducing NEMO-IKK supramolecular structures. J. Cell Biol. 2014; Vol. 204 No. 2: 231-245.
11. Kishida S, Sanjo H, Akira S, Matsumoto K, Ninomiya-Tsuji J. TAK1-binding protein 2 facilitates ubiquitination of TRAF6 and assembly of TRAF6 with IKK in the IL-1 signaling pathway. Genes Cells. 2005 May; 10 (5): 447-454.
12. Gao Q, Liu Y, Wu Y, Zhao Q, Wang L, Gao S et al. IL-17 intensifies IFN-γ-induced NOS2 upregulation in RAW264.7 cells by further activating STAT1 and NF-κB. Int J Mol Med. 2016; 37: 347 – 358.
13. Chan ED, Winston BW, Uh ST, Wynes MW, Rose DM, Riches DWH. Evaluation of the role of mitogen-activated protein kinases in the expression of inducible nitric oxide synthase by IFN-γ and TNF-α in mouse macrophages. J Immunol. 1999; 162: 415 – 422.
14. Deb A, Haque SJ, Mogensen T, Silverman RH, Williams BRG. RNA-Dependent protein kinase PKR is required for activation of NF-κB by IFN-γ in a STAT1-independent pathway. J Immunol. 2001; 166: 6170-6180.
15. Werner F, Jain MK, Feinberg MW, Sibinga NES, Pellacani A, Wiesel P et al. Transforming Growth Factor-β1 Inhibition of Macrophage Activation is Mediated via Smad3. J Biol Chem. 2000; 275(47):36653-36658.
16. Ramana CV, Chatterjee-Kishore M, Nguyen H, Stark GR. Complex roles of Stat1 in regulating gene expression. Oncogene. 2000; 19(21): 2619-27.
17. Xiong H, Zhu C, Li H, Chen F, Mayer L, Ozato K et al. Complex formation of the interferon (IFN) consensus sequence-binding protein with IRF-1 is essential for murine macrophage IFN-γ-induced iNOS gene expression. J Biol Chem. 2003; Vol 278, No. 4: 2271 – 2277.
18. Chesrown SE, Monnier J, Visner G, Nick HS. Regulation of inducible nitric oxide synthase mRNA levels by LPS, INF-γ, TGF-β, and IL-10 in murine macrophage cell lines and rat peritoneal macrophages. Biochem. Biophys. Res. Commun. 1994; Vol 200 No.1: 126 – 134.
19. Takaki H, Minoda Y, Koga K, Takaesu G, Yoshimura A, Kobayashi T. TGF-β1 suppresses IFN-γ-induced NO production in macrophages by suppressing STAT1 activation and accelerating iNOS protein degradation. Genes Cells. 2006; 11(8):871-882.
20. Burke SJ, Updegraff BL, Bellich RM, Goff MR, Lu D, Minkin Jr SC et al. Regulation of iNOS gene transcription by IL-1β and IFN-γ requires a coactivator exchange mechanism. Mol Endocrinol. 2013 October; 27(10): 1724-1742.
21. Zhu X, Wen Z, Xu LZ, Darnell Jr JE. STAT1 serine phosphorylation occurs independently of tyrosine phosphorylation and requires an activated Jak2 kinase. Mol Cell Biol. 1997; Vol 19, No. 11: 6618 – 6623.
22. Nguyen H, Chatterjee-Kishore M, Jiang Z, Qing Y, Ramana CV, Bayes J et al. IRAK-dependent phosphorylation of Stat1 on serine 727 in response to interleukin-1 and effects on gene expression. J Interferon Cytokine Res. 2003; 23: 183-192.
23. Luu K, Greenhill CJ, Majoros A, Decker T, Jenkins BJ, Mansell A. STAT1 plays a role in TLR signal transduction and inflammatory responses. Immunol Cell Biol. 2014; 1-9.
24. Lee S, Nishino M, Mazumdar T, Garcia GE, Galfione M, Lee FL et al. 16-kDa prolactin down-regulates inducible nitric oxide synthase expression through inhibition of the signal transducer and activator of transcription 1/ IFN regulatory factor-1 pathway. Cancer Res. 2005; 65: (17).
25. Kovarik P, Stoiber D, Eyers PA, Menghini R, Neininger A, Gaestel M et al. Stress-induced phosphorylation of Stat1 at ser727 requires p38 mitogen –activated protein kinase whereas IFN-γ uses a different signaling pathway. PNAS. 1999; Vol 96, No 24: 13956 – 13961.
26. Lo H, Hsu S, Ali-Syed M, Gunduz M, Xia W, Wei Y et al. Nuclear interaction of EGFR and STAT3 in the activation of the iNOS/NO pathway. Cancer Cell. 2005; Vol. 7: 575 – 589.
27. Yu Z, Zhang W, Kone BC. Signal transducers and activators of transcription 3 (STAT3) inhibits transcription of the inducible nitric oxide synthase gene by interacting with nuclear factor κB. Biochem. J. 2002; 367, 97 – 105.
28. Müller-Ladner U, Judex M, Ballhorn W, Kullmann F, Distler O, Schlottmann K et al. Activation of IL-4 STAT pathway in Rheumatoid Synovium. J Immunol. 2000; 164: 3894-3901.
29. Ohmori Y, Hamilton TA. Interleukin-4/STAT6 represses STAT1 and NF-κB-dependent transcription through distinct mechanisms. J Biol Chem. 2000; 275(48): 38095-38103.
30. Xu D, Qu CK. Protein tyrosine phosphatases in the JAK/STAT pathway. Front Biosci. 2008; 13: 4925-4932.
31. Borderie D, Hilliquin P, Hernvann A, Lemarechal H, Kahan A, Menkes CJ et al. Inhibition of inducible NO Synthase by TH2 cytokines and TGF-β in rheumatoid arthritic synoviocytes: effects on Nitrosothiol production. Nitric Oxide. 2002; 6(3), 271-282.
32. Sugiyama Y, Kakoi K, Kimura A, Takada I, Kashiwagi I, Wakabayashi Y et al. Smad2 and Smad3 are redundantly essential for the suppression of iNOS synthesis in macrophages by regulating IRF3 and STAT1 pathways. Int Immunol. 2012; 24(4):253-265.
33. Pohlers D, Beyer A, Koczan D, Wilhelm T, Thiesen HJ, Kinne RW. Constitutive upregulation of the transforming growth factor-β pathway in rheumatoid arthritis synovial fibroblasts. Arthritis Res Ther. 2007; 9(3): R59.
34. Berg DT, Gupta A, Richardson MA, O’Brien LA, Calnek D, Grinnell BW. Negative regulation of inducible nitric-oxide synthase expression mediated through transforming growth factor-β- dependent modulation of transcription factor TCF11. J.Biol.Chem. 2007; Vol. 282, No. 51: 36837 – 36844.
35. Xu W, Comhair SAA, Zheng S, Chu SC, Marks-Konczalik J, Moss J et al. STAT-1 and c-Fos interaction in nitric oxide synthase-2 gene activation. Am J Physiol Lung Cell Mol Physiol. 2003; 285: L137-L148.
36. Xu G, Zhang Y, Zhang L, Roberts AI, Shi Y. C/EBPβ mediates synergistic upregulation of gene expression by IFNγ and TNFα in bone marrow-derived mesenchymal stem cells. Stem Cells. 2009 April; 27(4): 942-948.
37. Teng X, Zhang H, Snead C, Catravas JD. Molecular mechanisms of iNOS induction by IL-1β and IFN-γ in rat aortic smooth muscle cells. Am J Physiol Cell Physiol. 2002; 282: C144-C152.
38. Park KS, Guo Z, Shao L, Du Q, Geller DA. A far upstream Oct-1 motif regulates cytokine-induced transcription of the human inducible nitric oxide synthase (hiNOS) gene. J Mol Biol. 2009 July; 390(4): 595-603.
39. Hiroi M, Sakaeda Y, Yamaguchi H, Ohmori Y. Anti-inflammatory cytokine interleukin-4 inhibits inducible nitric oxide synthase gene expression in the mouse macrophage cell line RAW264.7 through the repression of octamer-dependent transcription. Mediators Inflamm. 2013; 2013: 369693.
